# On the role of extrinsic noise in microRNA-mediated bimodal gene expression

**DOI:** 10.1371/journal.pcbi.1006063

**Published:** 2018-04-17

**Authors:** Marco Del Giudice, Stefano Bo, Silvia Grigolon, Carla Bosia

**Affiliations:** 1 Department of Applied Science and Technology, Politecnico di Torino, Torino, Italy; 2 Italian Institute for Genomic Medicine, Torino, Italy; 3 Nordita, Royal Institute of Technology and Stockholm University, Stockholm, Sweden; 4 The Francis Crick Institute, London, United Kingdom; University of Basel, SWITZERLAND

## Abstract

Several studies highlighted the relevance of extrinsic noise in shaping cell decision making and differentiation in molecular networks. Bimodal distributions of gene expression levels provide experimental evidence of phenotypic differentiation, where the modes of the distribution often correspond to different physiological states of the system. We theoretically address the presence of bimodal phenotypes in the context of microRNA (miRNA)-mediated regulation. MiRNAs are small noncoding RNA molecules that downregulate the expression of their target mRNAs. The nature of this interaction is titrative and induces a threshold effect: below a given target transcription rate almost no mRNAs are free and available for translation. We investigate the effect of extrinsic noise on the system by introducing a fluctuating miRNA-transcription rate. We find that the presence of extrinsic noise favours the presence of bimodal target distributions which can be observed for a wider range of parameters compared to the case with intrinsic noise only and for lower miRNA-target interaction strength. Our results suggest that combining threshold-inducing interactions with extrinsic noise provides a simple and robust mechanism for obtaining bimodal populations without requiring fine tuning. Furthermore, we characterise the protein distribution’s dependence on protein half-life.

## Author summary

Phenotypic differentiation often relies on bimodal distributions of gene expression levels, which can normally be achieved by different molecular mechanisms. During the past decade microRNAs, small noncoding RNA molecules, were found to downregulate the expression of preferred mRNA targets by sequestering and successively degrading them, thus influencing the level of gene expression. We theoretically address the question on how microRNA-mediated regulation can induce the appearance of bimodal phenotypes. Our findings show that the presence of extrinsic noise favours bimodal distributions. This suggests a simple mechanism for obtaining bimodal populations where the presence of extrinsic noise relaxes the requirements on parameters fine tuning.

## Introduction

Gene-expression data displays bimodal distributions in several systems ranging from cancer to immune cells [1, 2]. The two peaks of the distribution are usually associated with different physiological states of the system, be that different stem-cell fates or different disease states or cancer subtypes [3–6]. From a theoretical point of view, a common belief is that bimodality is directly related to bistability, i.e., to multiple steady states appearing in the absence of noise. However, as shown in [7] and reviewed in [8], bimodality in some biological systems is solely due to stochastic effects. Environmental fluctuations, usually referred to as extrinsic noise [9, 10], can be a source of noise in molecular networks. Together with intrinsic fluctuations due to the probabilistic nature of chemical reactions, extrinsic noise shapes gene expression and may in principle drive cell differentiation.

In the past, both theoretical [11, 12] and in vitro [13] studies, have highlighted the possibility that microRNAs (miRNAs), in particular stoichiometric conditions, may induce bimodality in the expression of their targets simply because of stochastic effects related to their specific titrative interactions and not because of a bistable system. This is similar to the action of miRNAs’ bacterial counterpart [14].

MiRNAs are small molecules of non-coding RNA, found in eukaryotes to act as post-transcriptional regulators. Although they were found in several different eukaryotic kingdoms, their role is known to be vital in multicellular organisms. They perform this function by recognising mRNA targets through Watson-Crick base pairing. Once bound to the target, they prevent its translation and can enhance its instability by degrading it. Interestingly, different levels of miRNA-target interactions can be achieved by different numbers of miRNAs [15–17]. Theoretical predictions [12] together with in vitro single-cell experiments [13] suggested that bimodality in the expression levels of miRNA targets can be achieved with a high miRNA-target interaction strength. In terms of genetic sequences, this would imply a high specificity between target and miRNA, and therefore a high number of complementary binding sites (bs) per target.

**Fig 1:**
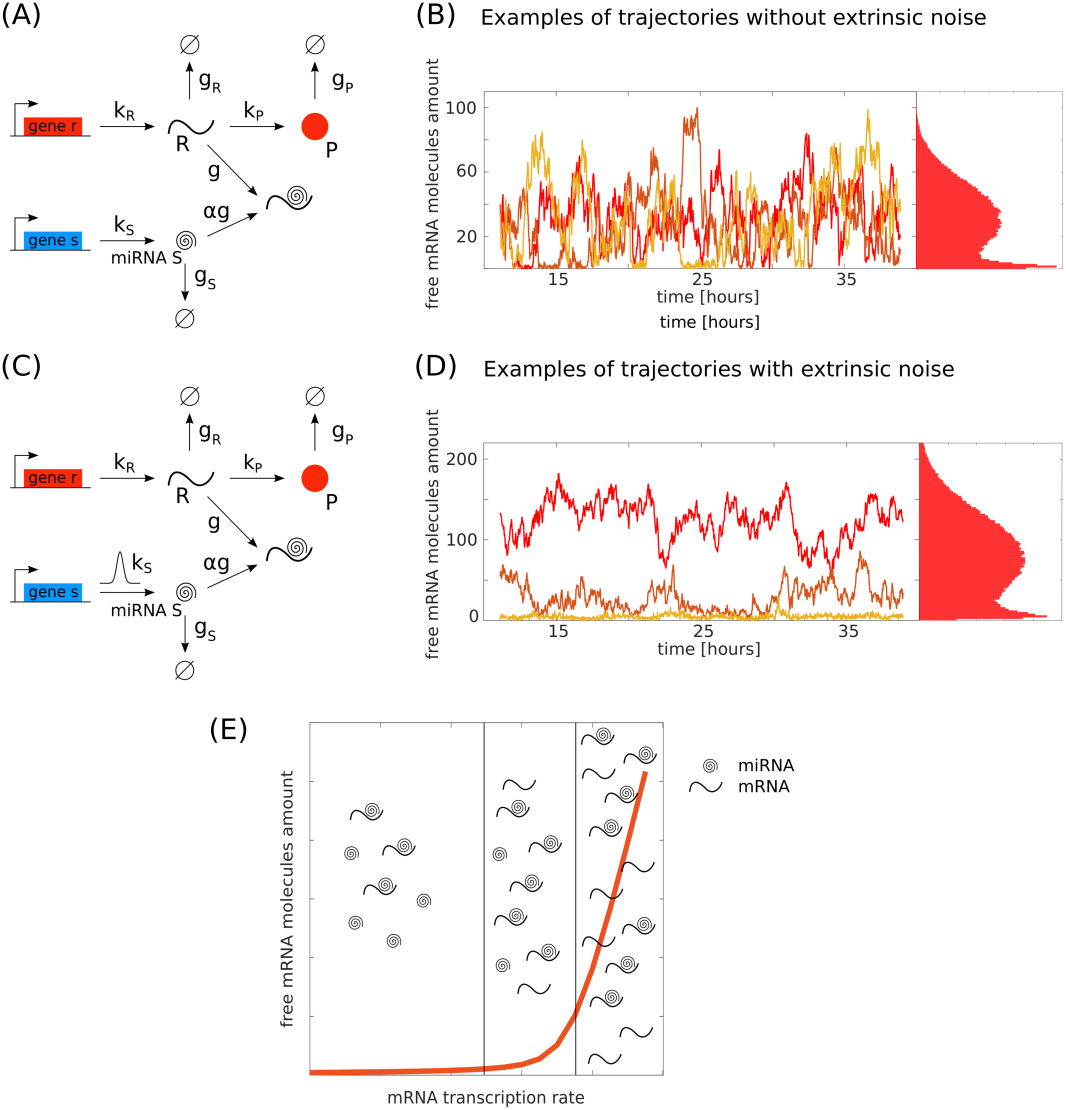
Model and steady-state trajectories. The reference circuits (with and without extrinsic noise on the miRNA transcription rate) are represented in (C) and (A) respectively with the rates considered in the model. *k*_*R*_ and *k*_*S*_ are the target mRNA and miRNA transcription rates, *g*_*R*_ and *g*_*S*_ are respectively the mRNA and miRNA degradation rates. *k*_*P*_ is the protein translation rate and *g*_*P*_ is its degradation rate. *g* is the miRNA-target interaction strength and *α* is the fraction of miRNAs that are not recycled after binding to the mRNA. In panels (B) and (D) there are three different trajectories for the mRNA, corresponding to the model on the left. For both panels, the steady-state distributions of the number of free mRNA molecules are bimodal. In (B) the parameters are *k*_*S*_ = 1.2 × 10^−3^ nM min^−1^, *k*_*R*_ = 2.7 × 10^−3^ nM min^−1^, *g*_*S*_ = 1.2 × 10^−2^ min^−1^, *g*_*R*_ = 2.4 × 10^−2^ min^−1^, *g* = 1.5 × 10^3^ nM^−1^ min^−1^, *k*_*P*_ = 6.0 min^−1^, *g*_*P*_ = 1.2 × 10^−2^ min^−1^ and *α* = 0.5. In (D) the parameters are *k*_*R*_ = 3.1 × 10^−3^ nM min^−1^, *g*_*S*_ = 1.2 × 10^−2^ min^−1^, *g*_*R*_ = 2.4 × 10^−2^ min^−1^, *g* = 1.2 × 10^2^ nM^−1^ min^−1^, *k*_*P*_ = 6.0 min^−1^, *g*_*P*_ = 1.2 × 10^−2^ min^−1^ and *α* = 0.5. *k*_*S*_ are picked from a Gaussian distribution with mean ${\bar{k}}_{S}=1.2\times 10^{-3}\;\text{nM}\;{\text{min}}^{-1}$ and standard deviation *σ* = 2.4 × 10^−4^ nM min^−1^. (E) Cartoon of the free mRNA threshold behaviour as a function of its transcription rate. Below the threshold the amount of free miRNA is greater then the amount of free mRNA, in proximity to the threshold their amount is nearly the same, above the threshold the free mRNA amount exceeds the miRNA.

As long as a miRNA molecule is bound to the target, it cannot be translated. It is then possible to define a threshold for the mRNA transcription rate such that below the threshold many of the mRNA target molecules are bound to miRNAs and above the threshold there are molecules of mRNA free for translation [12, 18, 19]. The titrative interaction between miRNA and mRNA thus induces a specific dependence between the amounts of the two species in the system. Such a threshold mechanism involves two regimes that can be explored by varying, e.g., the mRNA transcription rate. The two regimes are characterized by a low and a high expression of mRNA. In more detail, the threshold behaviour of mRNA expression crucially depends on three main parameters: the transcription rate of miRNA, the transcription rate of mRNA, and the interaction strength between miRNA and mRNA, which is related to the affinity between them and to the number of miRNA binding sites on the target. If the amount of miRNA is high compared to that of mRNA, mRNA will be sequestered and repressed. Conversely, if the quantity of miRNA is low compared to the mRNA, mRNA will be expressed and free for translation. The two regimes can be explored by varying the parameters. For instance, starting with very few free molecules of mRNA and gradually increasing the mRNA transcription rate moves the system from the repressed to the expressed state as shown in Fig 1E. The transition from repressed to expressed upon increasing the mRNA transcription rate occurs in a non-linear fashion when the amount of mRNA molecules roughly matches that of miRNAs. The steepness of the threshold at this point depends on the interaction strength between miRNA and mRNA. The presence of the threshold was first detected in [18] where the mechanism described above was shown and explained in terms of a titrative interaction. The dependence of the threshold location on miRNA expression was investigated in two different papers, namely in [13] for the highly expressed miRNA regime and in [20] for the weakly expressed miRNA regime.

Close to the threshold the number of both free miRNAs and targets is small. Their fluctuations are highly coupled by the non-linear interaction between the two and a small fluctuation in their amounts may lead the system from the “bound” to the “unbound” state [12, 13, 19].

As anticipated, if the interaction strength between miRNA and target is high, then the transition from the bound to the unbound state is sharp. Close to the threshold, a subset of the targets will be bound to the miRNA and a subset will be unbound. This is because of the intrinsic fluctuations in the amount of both miRNA and target. Picturing this in terms of the target distribution would lead to a bimodal distribution whose two modes are associated with the bound and unbound state. It is worth underlining that this kind of bimodality is due to the presence of noise and not to peculiar molecular mechanisms introducing multiple deterministic stable states in the system.

MiRNAs are predicted to regulate more than 60% of our genome through a combinatorial action: every single miRNA can regulate several targets and one target can be regulated by different miRNAs [17, 21]. The variety of targets they regulate is so wide and important for different signalling pathways or developmental stages [22, 23] that the alteration of their expression levels is thought to contribute to tumour development and metastatisation [24–27]. As discussed in [28], a role for miRNA in generating expression variability can be remarkable. If miRNA activity increases the cell-to-cell variability of pivotal pluripotency factors, (consistent with observations in [29]), and in turn of pluripotency networks, then miRNA expression variability can provide an efficient mechanism for generating transitions between cell states.

Although extrinsic noise may influence gene expression and regulation at different levels, large variability across a cell population seems to be dominated by the population dynamics [30]. Even a monoclonal population has cells in different phases of their cell cycle because of growth and divisions. It is nowadays well established that multiple cell-cycle regulators are controlled by miRNAs, whose regulation could be in turn cell-cycle dependent [31–34]. The expression level of miRNAs may thus change with cell-cycle progression, and there are indeed miRNAs differentially expressed according to the particular phase of the cell cycle [35]. As a consequence, in a population of cells heterogeneous with respect to the cell cycle, such as non-quiescent cancer cells, the amount of miRNAs can strongly fluctuate from cell to cell. This introduces an extra source of noise in the system besides the intrinsic stochasticity of chemical reactions involving gene transcription, translation and regulation.

The aim of this work is to understand, with the aid of analytics and numerics, how extrinsic noise on miRNA expression can induce bimodality on the miRNA targets. Of course, such bimodality is not omnipresent but restricted to particular stoichiometric conditions and levels of noise. We show how a distribution of miRNA transcription rates reshapes the threshold between miRNA and target and defines a wider region of bimodality compared to case without extrinsic noise. Such a bimodal distribution can be seen at a “population level”, since the amount of miRNA is heterogenous throughout the different cells. This outcome is significantly different from previous results where differential phenotypic expression is induced by the strong coupling between miRNA and its target at the “single-cell level”. We also show that, if the miRNA target is protein coding, the protein half-life can alter the protein distribution. With respect to the shape of the mRNA distribution, an increased protein half-life leads to a narrowing of the protein distribution around its mean. This may promote or suppress bimodality, suggesting that bimodal distributions at the level of mRNA may still correspond to a specific single phenotype at the protein level. Conversely, repressed heavy tailed mRNA distributions may give rise to bimodal protein distributions.

Finally, given the existence of multiple targets competing for one type of miRNA, we ask whether these properties can be maintained in a more complex circuit made of two competing endogenous RNAs (ceRNAs) and one miRNA [36]. The different target genes indeed act as sponges for the miRNA molecules and may sequester them from the environment. As a result, the overexpression or underexpression of one of the targets can lead respectively to an increase or decrease in the level of expression of the other competitors. The intensity of such cross regulation depends on the distance from the threshold of quasi equimolarity between miRNAs and targets [12, 19]. This suggests that, if one target has a bimodal distribution, such bimodality may be influenced by the expression levels of the other miRNA competitors.

## Materials and methods

### Stochastic model for miRNA-target interaction with extrinsic noise

Models of microRNA-mediated circuits have been the subject of several recent studies [18, 37–41]. Here we will describe one of the simplest ways of accounting for microRNA-driven inhibition, depicted in Fig 1A. The molecular species involved in this circuit are miRNAs (S), target mRNAs (R) and proteins (P), resulting from the translation of the target mRNA.

In the following, we shall assume miRNAs and mRNAs are transcribed from independent genes. For simplicity we neglect all the intermediate reactions leading to the synthesis of mRNAs, therefore assuming they are produced at constant rate *k*_*R*_. For the miRNA, we consider it to be transcribed with a constant rate *k*_*S*_ which we let fluctuate between different cells to probe the effects of extrinsic noise on the system. This approach is equivalent to having *k*_*S*_ slowly fluctuating in time with respect to the systems’ reactions while different cells are stochastically unsynchronised. We also remark that, in the opposite limit of very rapid extrinsic fluctuations, these variations average out and the system effectively behaves as if subject to intrinsic noise only. A more detailed analysis of the interplay between the time scales of extrinsic fluctuations and those of the system is reported in a devoted section of the Results. MiRNAs and mRNAs can also be degraded by the action of specialised enzymes. Here we assume these reactions are governed by mass-action laws with rates *g*_*S*_ and *g*_*R*_. The associated molecular reactions read:

$$\emptyset \overset{k_{R}}{\underset{g_{R}}{⇌}}R,\;\emptyset \overset{k_{S}}{\underset{g_{S}}{⇌}}S.$$ (1)

MiRNAs act as post-transcriptional regulatory elements, by binding the target mRNAs in a complex that can be subsequently degraded. Such interactions between miRNAs and mRNAs are quantified by the effective parameter *g*, which takes into account the strength of the miRNA-target coupling: from a biochemical point of view, it depends on the affinity between the two molecular species and on the number of miRNA binding sites dedicated to a specific target [18]. The formation of the miRNA-mRNA complex reads:

$$\begin{array}{c} R+S\overset{g}{⟶}RS. \end{array}$$ (2)

While the mRNAs are always degraded due to the titrative interaction, the miRNAs can be recycled with probability 1 − *α* in the following way:

$$\begin{array}{c} RS\overset{1-\alpha }{⟶}S. \end{array}$$ (3)

Whenever the mRNAs are not bound to miRNAs, they can be translated into proteins with translation rate *k*_*P*_ and, as assumed for the other molecular species, proteins can also be degraded with rate *g*_*P*_, i.e.:

$$\begin{array}{c} R\overset{k_{P}}{⟶}R+P,\;\;P\overset{g_{P}}{⟶}\emptyset . \end{array}$$ (4)

From now on, we define as “intrinsic noise” the fluctuations due to the stochasticity of the chemical reactions with constant rates (Fig 1A) and as “extrinsic noise” those due to the fluctuating miRNA transcription rate (see Fig 1C).

The system can be described by the probability distribution $P(n_{S},n_{R},n_{P},t|K)$ of observing *n*_*S*_ molecules of miRNA, *n*_*R*_ molecules of mRNA and *n*_*P*_ proteins at time *t* given a set of parameters $K=\{k_{R},k_{S},k_{P},g_{R},g_{S},g_{P},g,\alpha \}$. This probability distribution follows the same master equation presented in [12] that can be either solved numerically or at the steady-state with some approximations. If the parameters fluctuate, this must be taken into account in order to obtain the full distribution at steady state *P*_*ss*_(*n*_*S*_, *n*_*R*_, *n*_*P*_). This can be achieved by using the law of total probability [42], which states that $P\left(n_{S},n_{R},n_{P}\right)=\int P\left(K\right)P\left(n_{S},n_{R},n_{P}|K\right)dK$. As our aim is to test the effects of a fluctuating miRNA transcription rate, we shall assume this to be the only parameter drawn from a probability distribution, specifically a Gaussian centred around 〈*k*_*S*_〉 with variance $\sigma_{k_{S}}^{2}$ and defined only for positive values of *k*_*S*_. As previously mentioned, this is equivalent to assuming the extrinsic noise fluctuates slowly compared to the typical time scale of any other reaction in the system.

To obtain the steady-state distribution *P*(*n*_*S*_, *n*_*R*_, *n*_*P*_|*k*_*S*_) conditional on a specific miRNA transcription rate we could choose different approximation methods. Pivotal examples are the Van Kampen [43] and the Gaussian approximations [12]. In the following we focused on the former one, leaving to the Supporting Information a comparison between the two methods (see S1 Fig). We therefore performed a system-size expansion, thus assuming the system distribution at fixed parameters to be Gaussian.

The marginal distribution *P*(*n*_*S*_, *n*_*R*_, *n*_*P*_) was then found by using the law of total probability, i.e., by performing a weighted average over all possible values of *k*_*S*_.

Finally, we applied the same approach when considering two targets interacting with the same miRNA (Fig 5A). In this case the conditional distribution is *P*(*n*_*S*_, *n*_*R*1_, *n*_*R*2_, *n*_*P*1_, *n*_*P*2_|*k*_*S*_) from which one can obtain the full distribution by integrating over the values of the miRNA transcription rate.

#### Analytic approach

The equations governing the dynamics of the system considered in Fig 1 are given by:

$$\begin{array}{c} \frac{dR}{dt}=k_{R}-g_{R}R-gRS\\ \frac{dS}{dt}=k_{S}-g_{S}S-g\alpha RS\\ \frac{dP}{dt}=k_{P}R-g_{P}P\;\;, \end{array}$$ (5)

where *R*, *S* and *P* are the concentrations of the three species involved in the circuit expressed in nanomolars and the parameters are the same as in Fig 1.

Because of the inherent stochastic nature of molecular reactions, intrinsic noise should be taken into account by defining the probability distribution of observing $\underline{n}=\left(n_{R},n_{S},n_{P}\right)$ molecules at time *t*, namely $P(\underline{n},t)$. The number of molecules of species *X*, *n*_*X*_, relates to the concentrations, *ρ*_*X*_, as *n*_*X*_ = *V*_*cell*_*ρ*_*X*_. The dynamics of this system can be rewritten in terms of the master equation, that reads:

$$\begin{array}{cc} & \frac{dP(\underline{n},t)}{dt}=k_{R}[P\left(n_{R}-1,t\right)-P\left(\underline{n},t\right)]+\\ & \frac{g_{R}}{V_{cell}}[\left(n_{R}+1\right)P\left(n_{R}+1,t\right)-n_{R}P\left(\underline{n},t\right)]+\\ & k_{S}[P\left(n_{S}-1,t\right)-P\left(\underline{n},t\right)]+\\ & \frac{g_{S}}{V_{cell}}[\left(n_{S}+1\right)P\left(n_{S}+1,t\right)-n_{S}P\left(\underline{n},t\right)]+\\ & \frac{k_{P}n_{R}}{V_{cell}}[P\left(n_{P}-1,t\right)-P\left(\underline{n},t\right)]+\\ & \frac{g_{P}}{V_{cell}}[\left(n_{P}+1\right)P\left(n_{P}+1,t\right)-n_{P}P\left(\underline{n},t\right)]+\\ & \frac{g\alpha }{V_{cell}^{2}}[\left(n_{S}+1\right)\left(n_{R}+1\right)P\left(n_{R}+1,n_{S}+1,t\right)-n_{S}n_{R}P\left(\underline{n},t\right)]+\\ & \frac{g\left(1-\alpha \right)n_{S}}{V_{cell}^{2}}[\left(n_{R}+1\right)P\left(n_{R}+1,t\right)-n_{R}P\left(\underline{n},t\right)]. \end{array}$$ (6)

Solving the master equation, or even determining the first and second moments of $P(\underline{n},t)$ might be a difficult task due to the non-linear terms appearing from the miRNA-mRNA interactions. Therefore, it is common practice to use approximations, particularly van Kampen’s system-size expansion [43].

The van Kampen system-size expansion relies on the assumption that the number of molecules of any species *X* can be split into two contributions, one due to the deterministic system, and the other due to the intrinsic noise in the system, namely:

$$\begin{array}{c} n_{X}=V_{cell}\rho_{X}+V_{cell}^{1/2}\xi_{X}, \end{array}$$ (7)

where *ξ*_*X*_ is a Gaussian-distributed variable with zero average. The cell volume, *V*_*cell*_, represents the system size and it is assumed to be sufficiently large. Rewriting the master equation using Eq 7 to leading order in V_cell_, one finds the probability distribution to be a Dirac delta function in terms of the number of molecules. Thus the deterministic Eq 5 are recovered. The next-to-leading order in the expansion leads to a linear Fokker-Planck like equation in terms of the noisy variables {*ξ*_*X*_}. The resulting probability distribution for the number of molecules is Gaussian, centred around the deterministic concentration *ϕ*_*X*_, with finite variance. By using this equation, one can compute the variances and the cross-correlations of the noisy variables, namely $\langle \xi_{X}^{2}\rangle$ and 〈*ξ*_*X*_ *ξ*_*Y*_〉 that can be related to the molecules’ variances and cross-correlations.

In the specific case of the circuit we analysed, the variances and cross-correlations are steady-state solutions of the following system:

$$\begin{array}{c} \dot{\left\langle \xi_{R}^{2}\right\rangle }=k_{r}-2g_{r}\left\langle \xi_{R}^{2}\right\rangle +g_{r}R-2\alpha gR\left\langle \xi_{S}\xi_{R}\right\rangle -2\alpha gS\left\langle \xi_{R}^{2}\right\rangle +\\ \alpha gSR-2\left(1-\alpha \right)gS\left\langle \xi_{R}^{2}\right\rangle +\left(1-\alpha \right)gRS-2\left(1-\alpha \right)gR\left\langle \xi_{S}\xi_{R}\right\rangle \\ \dot{\left\langle \xi_{S}^{2}\right\rangle }=k_{s}-2g_{s}\left\langle \xi_{S}^{2}\right\rangle +g_{s}S-2\alpha gR\left\langle \xi_{S}^{2}\right\rangle -2\alpha gS\left\langle \xi_{S}\xi_{R}\right\rangle +\alpha gRS\\ \dot{\left\langle \xi_{P}^{2}\right\rangle }=k_{p}R-2g_{p}\left\langle \xi_{P}^{2}\right\rangle +g_{p}P+2k_{p}\left\langle \xi_{P}\xi_{R}\right\rangle \\ \dot{\left\langle \xi_{R}\xi_{S}\right\rangle }=-g_{r}\left\langle \xi_{S}\xi_{R}\right\rangle -g_{s}\left\langle \xi_{S}\xi_{R}\right\rangle -\alpha gR\left\langle \xi_{S}^{2}\right\rangle -\alpha gS\left\langle \xi_{S}\xi_{R}\right\rangle -\\ \alpha gR\left\langle \xi_{S}\xi_{R}\right\rangle -\alpha gS\left\langle \xi_{R}^{2}\right\rangle +RS\alpha g-\left(1-\alpha \right)gS\left\langle \xi_{S}\xi_{R}\right\rangle -\\ \left(1-\alpha \right)gR\left\langle \xi_{S}^{2}\right\rangle \\ \dot{\left\langle \xi_{R}\xi_{P}\right\rangle }=-g_{P}\left\langle \xi_{P}\xi_{R}\right\rangle +k_{P}\left\langle \xi_{R}^{2}\right\rangle -\alpha gR\left\langle \xi_{P}\xi_{S}\right\rangle -\alpha gS\left\langle \xi_{P}\xi_{R}\right\rangle -\\ \left(1-\alpha \right)gS\left\langle \xi_{P}\xi_{R}\right\rangle -\left(1-\alpha \right)gR\left\langle \xi_{P}\xi_{S}\right\rangle -g_{r}\left\langle \xi_{P}\xi_{R}\right\rangle \\ \dot{\left\langle \xi_{S}\xi_{P}\right\rangle }=-g_{P}\left\langle \xi_{P}\xi_{S}\right\rangle -g_{s}\left\langle \xi_{P}\xi_{S}\right\rangle +k_{P}\left\langle \xi_{S}\xi_{R}\right\rangle -\alpha gR\left\langle \xi_{P}\xi_{S}\right\rangle -\\ \alpha gS\langle \xi_{P}\xi_{R}\rangle \;\;. \end{array}$$ (8)

Using the expressions obtained from the previous system, specifying all the parameters, one can compute the probability distribution of the system, $P(\underline{n},t)$.

To investigate the role of environmental fluctuations (i.e. extrinsic noise) in our system of interest, we consider a fluctuating rate of miRNA production *k*_*S*_. For the sake of simplicity, we assume this parameter to be drawn from a Gaussian distribution, $P\left(k_{S}\right)=\frac{1}{\sqrt{2\pi \sigma_{k_{S}}^{2}}}e^{-\frac{\left(k_{S}-\langle k_{S}\rangle \right)}{2\sigma_{k_{S}}^{2}}}$, defined for positive values of *k*_*S*_, where 〈*k*_*S*_〉 and $\sigma_{k_{S}}^{2}$ are the average and variance of *k*_*S*_, respectively.

Due to the further stochasticity introduced by the extrinsic noise, the master equation previously derived no longer holds. Fluctuations on the parameter *k*_*S*_ do not allow us to rewrite a similar equation for this system in a simple way. However, as shown in [42], the probability distribution of the entire system, $P(\underline{n})$ can be rewritten in terms of conditional probabilities by using the law of total probability in the following way:

$$\begin{array}{c} P\left(\underline{n}\right)=\int P\left(k_{S}\right)P\left(\underline{n}|k_{S}\right)dk_{S}, \end{array}$$ (9)

where *P*(*k*_*S*_) is the Gaussian distribution in *k*_*S*_ and $P(\underline{n}|k_{S})$ is the conditional probability of observing a certain configuration of the system, $\underline{n}$, given a specific value of *k*_*S*_. This probability distribution is a solution of the master equation Eq 6 for any given *k*_*S*_. One can therefore again apply the van Kampen expansion on the master equation of $P(\underline{n}|k_{S})$ and obtain all the moments of this distribution (these will all be functions of the fluctuating parameter *k*_*S*_). The full solution can be obtained by averaging the result over all the values of *k*_*S*_.

#### Molecular simulations of microRNA mediated circuits via the Gillespie algorithm

Stochastic simulations have been performed by implementing the Gillespie direct algorithm [44]. All the results presented in this paper are obtained at the steady state.

#### Parameter setting

The parameters used to illustrate our results explore different ranges of experimentally measured values. While the mRNA half-lives, with typical values of 5−10 hours [45, 46], may range from a few minutes to about a day [47, 48], protein lifetimes normally vary between minutes and several days [49]. The variability of miRNA half-lives is large: many miRNAs expressed in the brain show a short half-life [50] although in most cases mature miRNAs are stable with half-lives that can span days [51, 52]. The parameter characterising the fraction of miRNAs recycled after the interaction with their targets, i.e. the degree of catalyticity *α* in the model, is poorly understood. Some studies support an almost complete catalyticity [53, 54], some others an almost complete stoichiometric interaction [52, 55, 56], with intermediate values reported in [57]. Hereby, we took a value of this parameter such that the mRNA amount is in the range of 10 − 1000 molecules per cell, in agreement with [58, 59], and the total amount of a given miRNA is well within 2000 molecules per cell, as measured in [13, 18] and inferred from [60]. All other parameters, when not varied, are of the same order of magnitude as those inferred in [39, 61]. Since the amount of extrinsic noise is an unknown quantity, we performed our analyses for values that give levels of fold repression of the target comparable with the experimentally measured ones [13, 18]. The fold repression, measured as the ratio between the constitutive expression of the target (i.e. the value the target would have in absence of miRNA regulation, when *g* → 0) and the target itself, ranges between 1 and 10.

## Results

### Single-cell versus population-induced bimodality

The understanding of bimodal distributions is usually related to cell-fate determination and differentiation. These mechanisms are at the basis of organism development and mis-development. It is therefore important to address the question of what might be the underlying molecular mechanisms allowing cell diversity and variability. Given the strong involvement of miRNAs in developmental decisions, we focus here on the miRNA network represented in Fig 1A, at the single-cell level. Previous works [18, 36] showed that the binding and unbinding reactions between miRNA and target give rise to non-trivial threshold effects in quasi equimolar conditions between miRNA and target (see Fig 1E) where the threshold is defined, in terms of miRNA and mRNA transcription rates, as $k_{S}^{*}=\alpha k_{R}^{*}$ [18]. If the miRNA is transcribed at a rate above the threshold value, $k_{S}>k_{S}^{*}$, the system is enriched in microRNA, which tends to bind most of the present mRNA and prevents its translation. In this regard, the system can be seen as below the threshold with respect to the target and we shall refer to it as in the “repressed state”. Above the threshold, the mean amount of free target increases linearly with its transcription rate. The scenario with free mRNA molecules will be denoted as the “unrepressed state” of the system. This threshold effect displaying a transition between the repressed and the unrepressed state gets more marked as the interaction strength between miRNA and targets increases. Close to the threshold value of the target transcription rate, due to the probabilistic nature of chemical reactions, the system will stochastically switch between the repressed and unrepressed state. Such stochastic switching is enough to give rise to bimodal target distributions which appear for a narrow range of the target transcription rate *k*_*R*_ [12] (Fig 1B). This bimodality characterises the single cell where the miRNA network is defined: every single cell can jump from the repressed to the expressed target state if the coupling constant with the miRNA is high enough.

**Fig 2:**
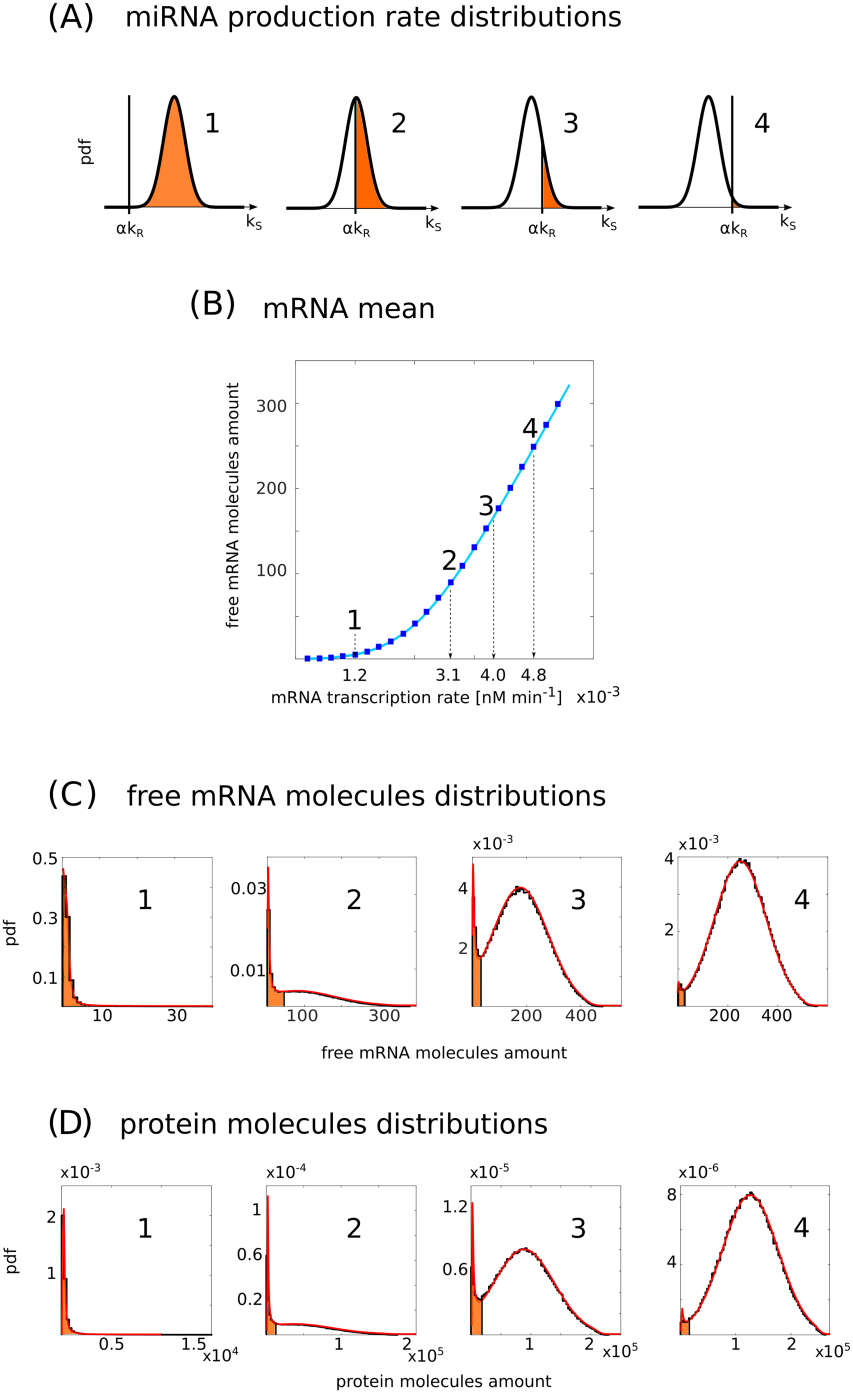
Emergence of bimodality in the presence of extrinsic noise. (A) Qualitative representation of the miRNA production rate distribution. The black vertical line indicates the value of the miRNA transcription rate *k*_*S*_ = *αk*_*R*_ for different values of the target transcription rate *k*_*R*_. The distributions represent the different conditions labelled from 1 to 4 shown in (B). The region of the distribution contributing to the repressed state is coloured in orange. (B) The amount of free mRNA molecules as a function of the target transcription rate *k*_*R*_. Solid lines are analytic predictions while blue squares correspond to numerical simulations. (C) Free mRNA molecule distributions corresponding to the points highlighted in (B). Solid black lines correspond to numerical simulations while solid red lines are analytic predictions. The repressed region is coloured in orange. (D) Protein molecule distributions corresponding to the mRNA distributions in (C). Solid black lines correspond to numerical simulations while solid red lines are analytic predictions. The repressed region is coloured in orange. In (B) the parameters are *g*_*S*_ = 1.2 × 10^−2^ min^−1^, *g*_*R*_ = 2.4 × 10^−2^ min^−1^, *g* = 1.2 × 10^2^ nM^−1^ min^−1^, *k*_*P*_ = 6.0 min^−1^, *g*_*P*_ = 1.2 × 10^−2^ min^−1^, *α* = 0.5. *k*_*S*_ are picked from a gaussian distribution with mean ${\bar{k}}_{S}=1.2\times 10^{-3}\;\text{nM}\;{\text{min}}^{-1}$ and standard deviation *σ* = 4.8 × 10^−4^ nM min^−1^. *k*_*R*_ ranges from 2.4 × 10^−4^ nM min^−1^ to 5.2 × 10^−3^ nM min^−1^. In (C) and (D) the parameters are the same as in (B) except for *k*_*R*_, which is fixed for each distribution and for dashed lines from 1 to 4 takes the values *k*_*R*_ = 1.2 × 10^−3^ nM min^−1^, 3.1 × 10^−3^ nM min^−1^, 4.0 × 10^−3^ nM min^−1^, 4.8 × 10^−3^ nM min^−1^.

**Fig 3:**
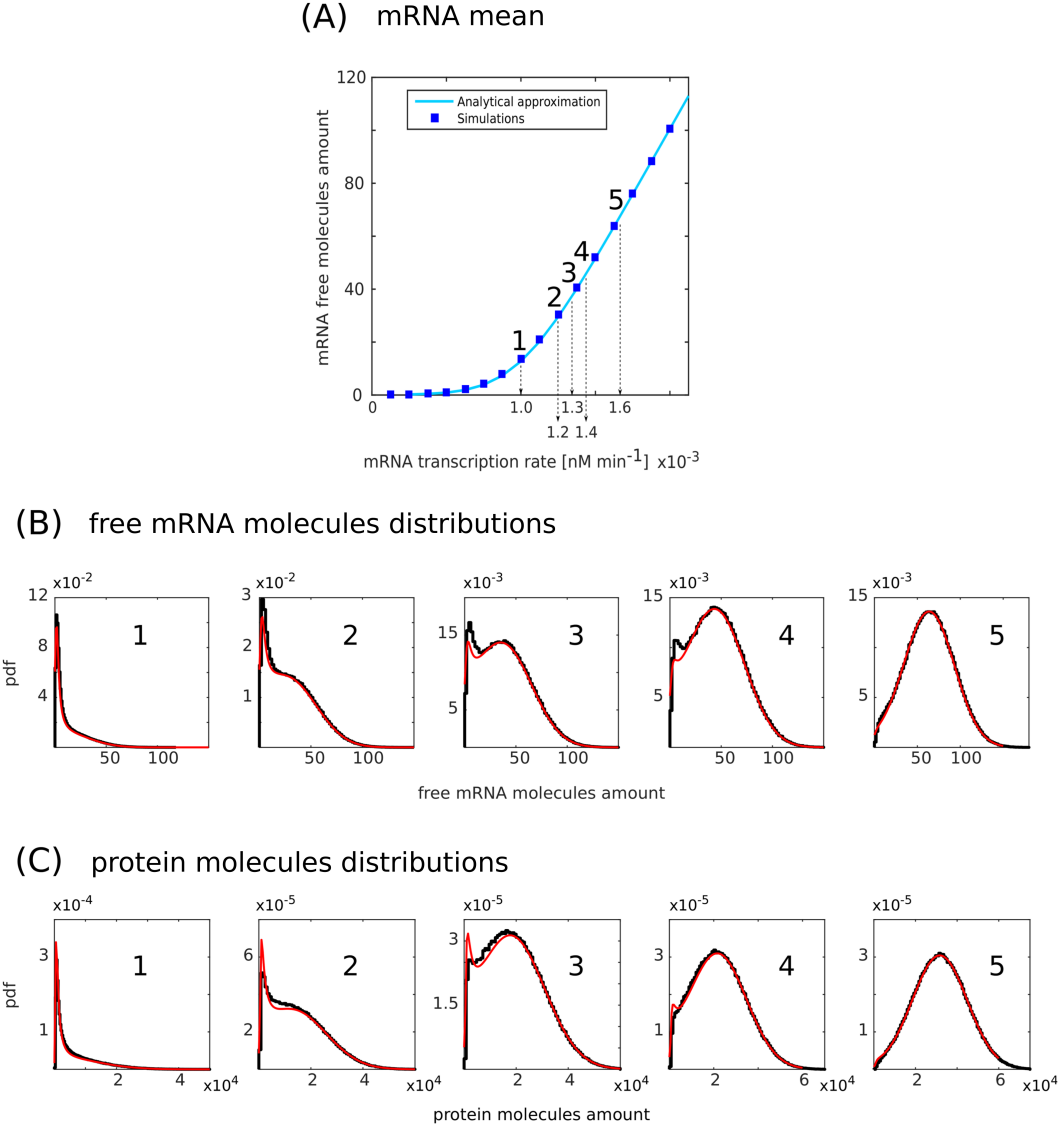
Bimodal distributions in the low-molecules regime. (A) Free mRNA molecules amount as a function of the target transcription rate *k*_*R*_. Solid lines are analytic predictions while blue squares correspond to numerical simulations. (B) Free mRNA molecules distributions corresponding to the conditions labelled from 1 to 5 in (A). Solid black lines correspond to numerical simulations while solid red lines are analytical predictions. (C) Protein molecules distributions corresponding to the mRNA distributions in (B). Solid black lines correspond to numerical simulations while solid red lines are analytical predictions. In (A) the parameters are *g*_*S*_ = 1.2 × 10^−2^ min^−1^, *g*_*R*_ = 2.4 × 10^−2^ min^−1^, *g* = 1.2 × 10^2^ nM^−1^ min^−1^, *k*_*P*_ = 6.0 min^−1^, *g*_*P*_ = 1.2 × 10^−2^ min^−1^, *α* = 0.5. *k*_*S*_ are picked from a gaussian distribution with mean ${\bar{k}}_{S}=4.8\times 10^{-4}\;\text{nM}\;{\text{min}}^{-1}$ and standard deviation *σ* = 1.2 × 10^−4^ nM min^−1^. *k*_*R*_ ranges from 1.2 × 10^−4^ nM min^−1^ to 19.2 × 10^−4^ nM min^−1^. In (B) and (C) the parameters are the same as in (A) except for *k*_*R*_ that is fixed for each distribution and from left to right takes the values *k*_*R*_ = 1.0 × 10^−3^ nM min^−1^, 1.2 × 10^−3^ nM min^−1^, 1.3 × 10^−3^ nM min^−1^, 1.4 × 10^−3^ nM min^−1^, 1.6 × 10^−3^ nM min^−1^.

**Fig 4:**
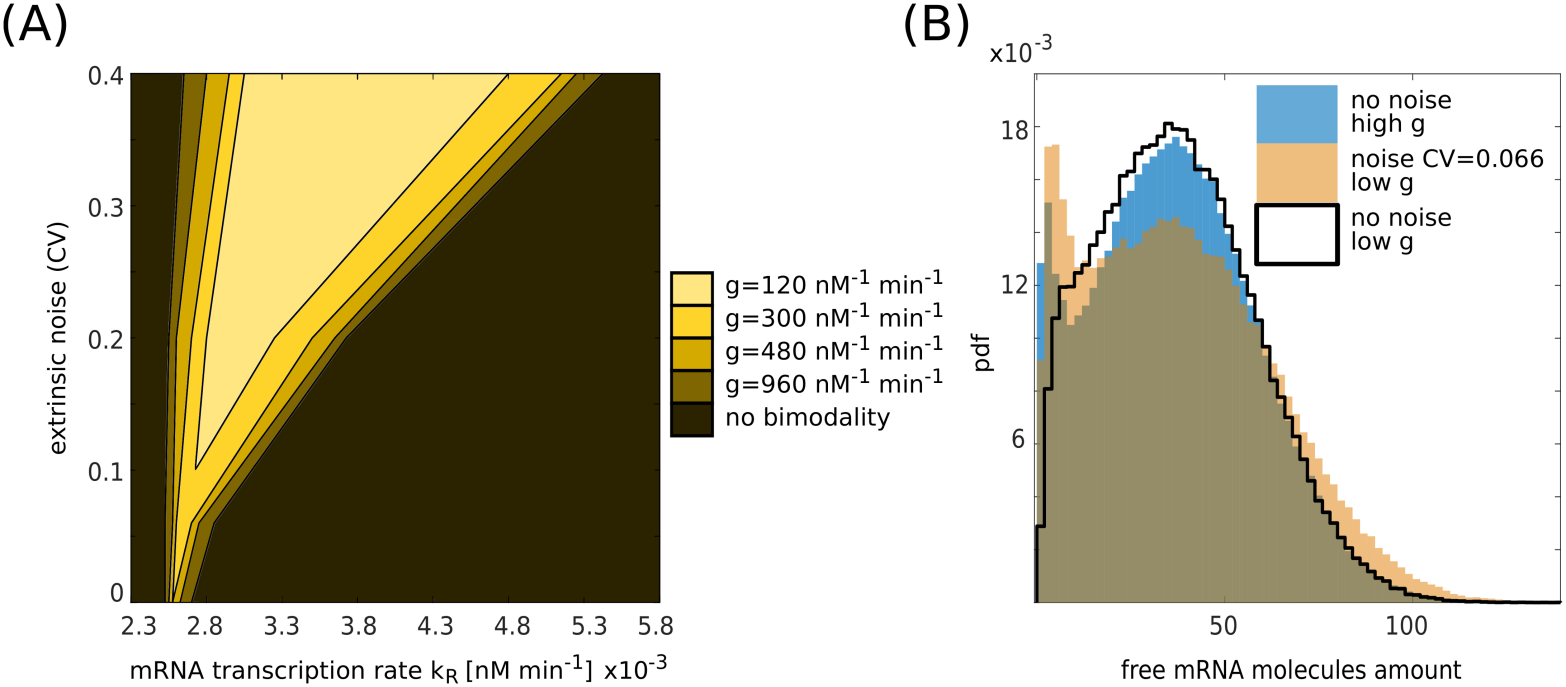
Bimodality as a function of the parameters. (A) Phase diagram for bimodality in the free mRNA molecules distribution. On the *x* axis there is the target transcription rate *k*_*R*_, on the *y* axis the extrinsic noise on the miRNA transcription rate. The color map indicates the presence of bimodality for different values of the miRNA-target interaction strength *g*. The presence of bimodality was computed by running several Gillespie’s simulations for fixed sets of parameters and sampling targets’ probability distributions. By using Matlab interpolation functions, we extracted the number of maxima of the distributions and used this value as a first measurement of bimodality. In the SI, we discussed a more refined version of this measurement. The width of the bimodality range increases as the interaction strength or the extrinsic noise are increased. The following parameters are equal for all the simulations: *g*_*S*_ = 1.2 × 10^−2^ min^−1^, *g*_*R*_ = 2.4 × 10^−2^ min^−1^, *k*_*P*_ = 6.0 min^−1^, *g*_*P*_ = 1.2 × 10^−2^ min^−1^, *α* = 0.5. Target mRNA transcription rate is one of the control parameters and ranges from *k*_*R*_ = 2.3 × 10^−3^ nM min^−1^ to *k*_*R*_ = 5.8 × 10^−3^ nM min^−1^. miRNA-mRNA association rate is one of the control parameters and takes the following values: *g* = 1.2 × 10^2^ nM^−1^ min^−1^, 3.0 × 10^2^ nM^−1^ min^−1^, 4.8 × 10^2^ nM^−1^ min^−1^, 9.6 × 10^2^ nM^−1^ min^−1^. Extrinsic noise is tuned by varying the standard deviation of the distribution with mean ${\bar{k}}_{S}=1.2\times 10^{-3}\;\text{nM}\;{\text{min}}^{-1}$ from which miRNA transcription rates are picked, the standard deviation takes the following values: *σ* = 0 nM min^−1^ (no extrinsic noise), 7.1 × 10^−5^ nM min^−1^, 2.4 × 10^−4^ nM min^−1^, 4.8 × 10^−4^ nM min^−1^. To define the origin of the bimodality region for the case with *g* = 1.2 × 10^2^ nM^−1^ min^−1^ the value *σ* = 1.2 × 10^−4^ nM min^−1^ was also used. In the SI, a more systematic non-binary study of the appearance of the bimodality is reported (see S5 Fig). (B) Free mRNA distribution in case of pure intrinsic noise and small (*g* = 3.8 × 10^2^ nM^−1^ min^−1^) miRNA-target interaction strength (black line), pure intrinsic noise and high (*g* = 1.1 × 10^3^ nM^−1^ min^−1^) miRNA-target interaction (blue histogram) and extrinsic noise (*σ* = 7.9 × 10^−5^ nM min^−1^) and small (*g*_1_ = 3.8 × 10^2^ nM^−1^ min^−1^) miRNA-target interaction strength (orange histogram). The other parameters are as follows: *k*_*S*_ = 1.2 × 10^−3^ nM min^−1^, *g*_*S*_ = 1.2 × 10^−2^ min^−1^, *k*_*R*_ = 2.7 × 10^−3^ nM min^−1^, *g*_*R*_ = 2.4 × 10^−2^ min^−1^, *k*_*P*_ = 6.0 min^−1^, *g*_*P*_ = 1.2 × 10^−2^ min^−1^, *α* = 0.5. The plot shows how extrinsic noise can compensate for small miRNA-target interaction strength in order to obtain bimodal distributions.

On the contrary, in presence of extrinsic noise, the miRNA transcription rate is not the same for every cell (Fig 1C and 1D). Hereby, we model the extrinsic noise through a Gaussian-distributed miRNA transcription rate. To understand intuitively the consequences of this kind of extrinsic noise, let us consider the case of a miRNA transcription-rate distribution with fixed average 〈*k*_*S*_〉. When the mRNA transcription rate (*k*_*R*_) is very low and the average miRNA transcription rate is much larger than the threshold value (〈*k*_*S*_〉 ≫ *αk*_*R*_), most of the drawn transcription rates *k*_*S*_ will be larger than the threshold value. This would place the network in the parameter range where the targets are almost all bound to the miRNAs (see Figs 2 and 3). For larger *k*_*R*_, approaching the threshold, values of *k*_*S*_ extracted from the left-tail will correspond to the case with some unbound targets. Below the threshold, as 〈*k*_*S*_〉 < *αk*_*R*_, the majority of the drawn *k*_*S*_ will belong to the unrepressed state with the right tail of the distribution sampling from the all-bound region (Figs 2A–2C and 3A and 3B). However, this scheme is the same as the previously mentioned population level scenario. The presence of rates above and below the threshold across the population can give rise to a bimodal distribution in the number of free targets (Figs 2C and 2D and 3B and 3C). In particular, the higher the extrinsic noise, the larger the range of target transcription rates for which bimodality is present and the greater the separation between the two phenotypes (bound and unbound targets), as depicted in Fig 4A. This implies that, in contrast to the case without extrinsic noise, it is no longer necessary to fine tune the transcription rates to obtain a bimodal distribution. Even for high values of *k*_*R*_, the fraction of randomly picked *k*_*S*_ that results in the bound state is not negligible and forms a visible peak in the distribution (Figs 2B–2D and 3A–3C). The bimodal distribution is in this case given by the superposition of unimodal distributions obtained for different *k*_*S*_ and weighted by the probability *P*(*k*_*S*_) (see S2 Fig in SI).

Focusing on one particular value of the variance $\sigma_{k_{S}}^{2}$ and varying the target transcription rate, *k*_*R*_, we monitored the appearance of bimodal distributions. We ran Gillespie’s simulations from which we sampled the number of targets for the histograms shown in Figs 2 and 3. By using system-size expansion and the law of total probability, we analytically obtained the target number distributions, shown in Figs 2 and 3. The analytic approximation captures the behaviour of the system for the mean, the Coefficient of Variation and the probability distribution, as testified by the agreement with the simulations (see Figs 2B–2D, 3A–3D and S3 in SI). In Fig 3 we showed that the results are maintained for a set of endogenously meaningful parameters, with low mean amount of free mRNAs. In this case, right because of the small amount of molecules involved, our approximation method is quantitatively less precise, though still keeping the qualitative shape of the distributions (see SI for details).

### A noisy environment can compensate for low miRNA-target interaction to obtain a bimodal distribution

To dissect the properties of bimodal distributions, we first ran Gillespie’s algorithm simulating the network in Fig 1C for different target transcription rates, *k*_*R*_, and different variances of the Gaussian noise on the miRNA transcription rates $\sigma_{k_{S}}^{2}$. Monitoring the appearance of bimodality, one can build up a phase diagram like the one shown in Fig 4A. In the absence of extrinsic noise but in the presence of intrinsic noise, bimodal distributions appear only for high coupling between the miRNA and the target and this region gets wider upon increasing the coupling constant *g*. Therefore the interaction strength between the miRNA and the target, *g*, affects the range of values of *k*_*R*_ in which bimodality is present.

Since in this case bimodality is a single-cell effect, only those cells having the target interacting strongly with the miRNA have a chance to experience the repressed and unrepressed state when *k*_*R*_ is close to its threshold value.

Adding some extrinsic noise relaxes the constraint on the interaction strength. Bimodality becomes a population effect, with some cells being locked in the repressed state (by having large miRNA transcription rates *k*_*S*_) and some others (with smaller *k*_*S*_) displaying free targets. Fig 4B shows how it is possible to have similar bimodal distributions either increasing the miRNA-target interaction strength (blue histogram) or increasing the extrinsic noise (orange histogram) with respect to a reference case with pure intrinsic noise and low miRNA-target interaction (black line). The extrinsic noise and the miRNA-target interaction strength act at a similar level with respect to bimodality, where a higher extrinsic noise can compensate for a low interaction strength (small number of miRNA binding sites on the target) in order to obtain two differentially expressed phenotypes.

### Interplay between different targets increases the stability of bimodal phenotypes

**Fig 5:**
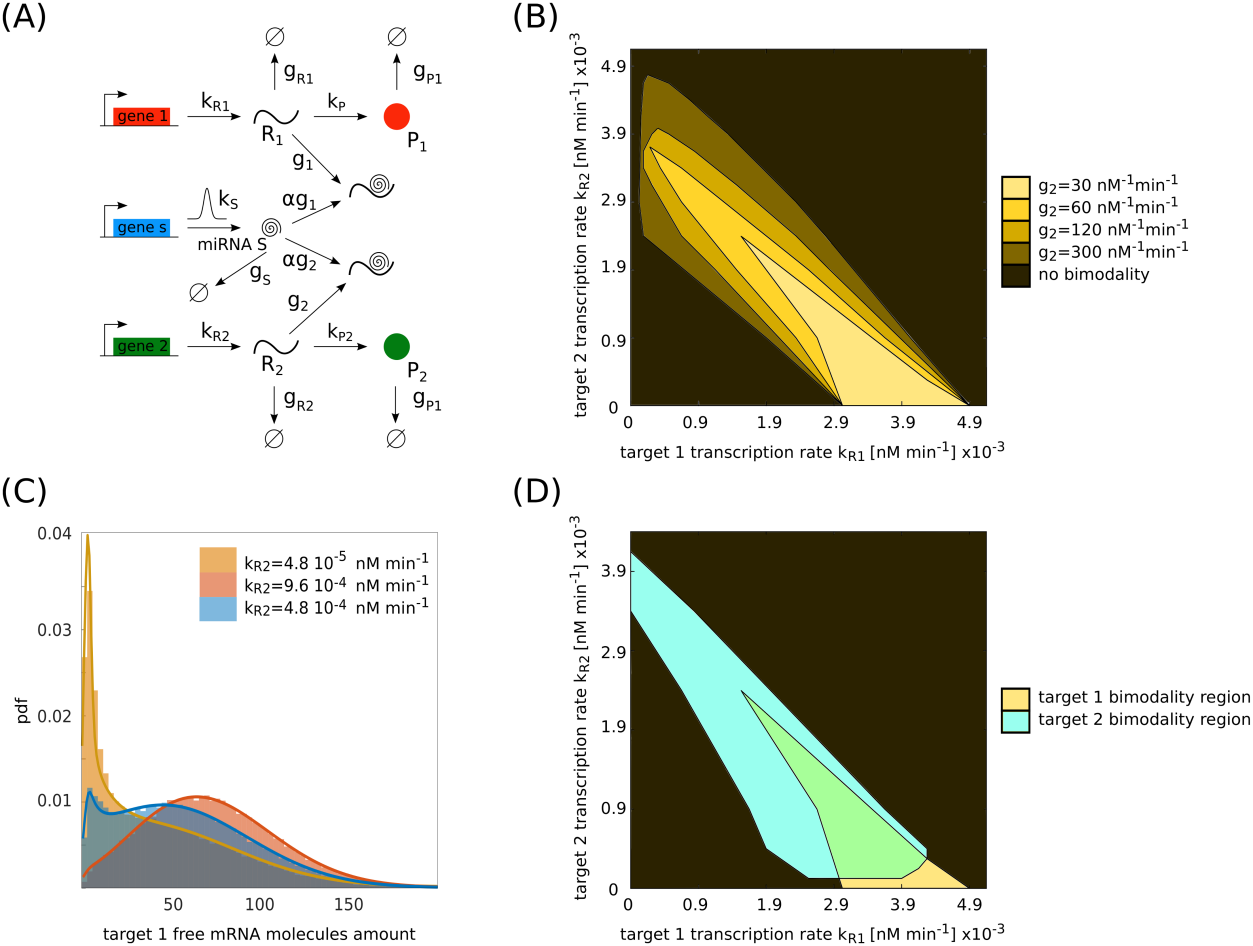
Competition between two targets of the same miRNA. (A) Reference circuit including extrinsic noise for the case of two genes competing for the same miRNA. *k*_*S*_ is the miRNA transcription rate. *k*_*R*1_ and *k*_*R*2_ are the mRNA transcription rates and *g*_*R*1_ and *g*_*R*2_ are the mRNA degradation rates of target 1 and 2 respectively. $k_{P_{1}}$ and *k*_*P*2_ are the translation rates and *g*_*P*1_ and *g*_*P*2_ are the degradation rates of protein 1 and 2 respectively. *g*1 and *g*2 are the miRNA interaction rates with target 1 and 2. *α* is the fraction of miRNAs that are not recycled after binding to the mRNAs. (B) Phase diagram for the bimodality of the target *R*_1_ for a fixed level of extrinsic noise (*σ* = 4.8 × 10^−4^ nM min^−1^), small miRNA/target 1 interaction strength (*g*_1_ = 1.2 × 10^2^ nM^−1^ min^−1^) and different miRNA/target 2 interaction strengths *g*_2_. Bimodality here has been measured as in Fig 4. The other parameters are as follows: *k*_*R*1_ and *k*_*R*2_ range from 0 nM min^−1^ to 5.1 × 10^−3^ nM min^−1^, ${\bar{k}}_{S}=1.2\times 10^{-3}\;\text{nM}\;{\text{min}}^{-1}$, *g*_*S*_ = 1.2 × 10^−2^ min^−1^, *g*_*R*1_ = *g*_*R*2_ = 2.4 × 10^−2^ min^−1^, *k*_*P*1_ = *k*_*P*2_ = 6.0 min^−1^, *g*_*P*1_ = *g*_*P*2_ = 2.4 × 10^−2^ min^−1^, *α* = 0.5. (C) Explanatory example of how it is possible to modulate target 1 distribution by increasing the expression of target 2 for small interaction strength between miRNA and targets (*g*_1_ = 1.2 × 10^2^ nM^−1^ min^−1^, *g*_2_ = 30 nM^−1^ min^−1^). The extrinsic noise here is *σ* = 2.4 × 10^−4^ nM min^−1^. The other parameters are as in (B). (D) Intersection between the bimodality phase diagrams of both targets for *g*_1_ = 1.2 × 10^2^ nM^−1^ min^−1^ and *g*_2_ = 30 nM^−1^ min^−1^. The other parameters are as in (B). Bimodality here is measured as in Fig 4. As a reference, with *g*_1_ = 1.2 × 10^2^ nM^−1^ min^−1^ and *g*_2_ = 30 nM^−1^ min^−1^, in the bimodality region of the distribution of target 1, the average number of mRNA molecules of target 1 ranges from 40 to 250 and the average number of mRNA molecules of target 2 ranges from 0 to 125. In the bimodality region of the distribution of target 2, the average number of mRNA molecules of target 1 ranges from 0 to 60 and the average number of mRNA molecules of target 2 ranges from 5 to 180.

The study so far led us to consider the possible importance of extrinsic noise in cell phenotypic variability. Given the existence of multiple miRNA-target networks, we now investigate how the results for the one-target case extend to the multiple-target one. Let us consider a minimal model with two targets, *R*_1_ and *R*_2_, competing for the same miRNA, *S* (Fig 5A). We start by investigating the effect of an increase in the expression of target *R*_2_ on target *R*_1_ with and without extrinsic noise. Upon increasing the transcription rate $k_{R_{2}}$ of target *R*_2_, the threshold of the target *R*_1_ shifts towards lower expression levels: the miRNAs are indeed sequestered by *R*_2_ and a lower amount of *R*_1_ is needed to overcome the threshold.

If *R*_1_ has a high interaction strength *g*_1_ with the miRNA, then the range of bimodality shifts towards lower expression levels as well. The width of the range of *R*_1_ bimodality is determined by the interaction strength *g*_2_ of the target *R*_2_ with the miRNA. If *g*_2_ ≫ *g*_1_, then the miRNAs are sequestered by the second target with such a high frequency that the net effect is a reduction in the amount of miRNAs available to target *R*_1_. This entails a shift not only of the $k_{R_{1}}$ threshold value but also of the range of bimodality. If *g*_2_ < *g*_1_, the second target *R*_2_ interacts with low frequency with the miRNA, *R*_1_ is slightly derepressed and the net effect on its bimodal distribution is a reduction of the range of transcription rates for which it is present. The emerging picture is that, for a given transcription rate $k_{R_{1}}$, it is possible to tune the distribution of target *R*_1_ from monomodal to bimodal and from unrepressed to repressed and vice versa via the expression of target *R*_2_.

The presence of extrinsic noise also makes such cross regulation possible for cases with lower miRNA-target interaction on both targets. In Fig 5B we show the bimodality phase diagram for *R*_1_ at a fixed interaction strength *g*_1_ between miRNA and target *R*_1_, and for a fixed level of extrinsic noise (see S4 Fig in SI for a different noise level). The interaction strength is such that in the case of pure intrinsic noise *R*_1_ does not show a bimodal distribution. As an explanatory example, Fig 5C shows that the peaks of *R*_1_ distribution can be tuned towards the repressed or the unrepressed case by decreasing or increasing the expression of a second target *R*_2_. Here, the two targets *R*_1_ and *R*_2_ are both coupled through the noisy miRNA with small interaction strengths. Also in this case, the same analytic approach as before gives good agreement between theory and simulations.

These observations suggest that even if the miRNA repression is low and diluted over a network of multiple targets, the noisy environment allows cross-regulation between ceRNAs at the population level (see Fig 5D, with the intersection between the bimodality phase diagrams for both targets for a fixed level of noise and miRNA interaction strengths).

### Protein stability and bimodal phenotypes

**Fig 6:**
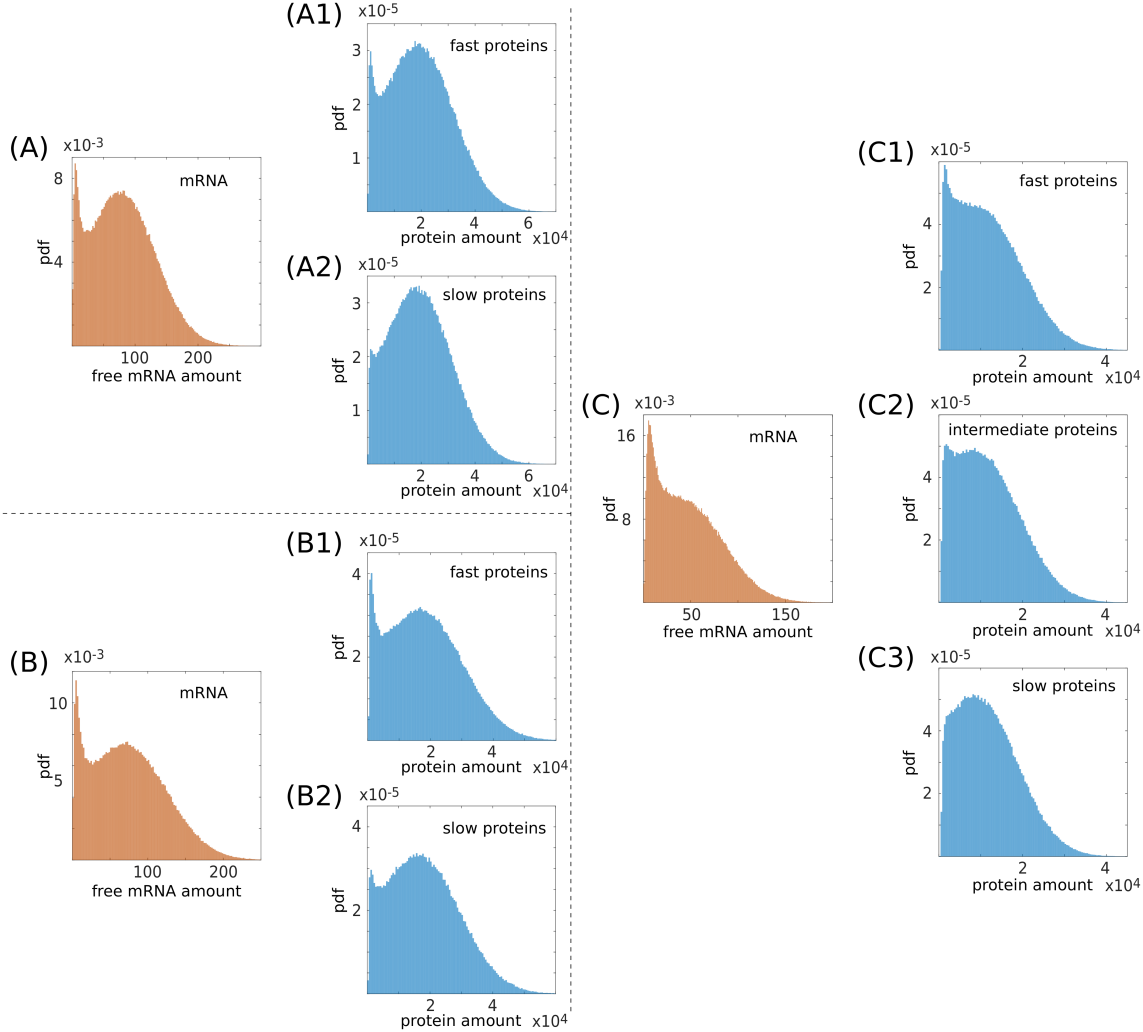
Protein half-life and bimodality. In this panel three conditions (A), (B) and (C) in which the shape of the protein distribution is altered by an increased protein stability are reported. Histograms are the result of numerical simulations. The free mRNA distributions are represented in orange, and the protein distributions in blue, corresponding to different levels of scale separation between the mRNA and protein dynamics. Fast protein distributions are obtained for a protein half life comparable to that of the mRNA; in this condition the state of the protein copies that of the mRNA and the distributions almost coincide. Slow protein distributions are obtained for a protein half life up to 10 times longer than that of the mRNA. As a consequence of the higher protein stability different outcomes can be achieved depending on the level of extrinsic noise, the miRNA-target interaction strength and the proximity to the threshold (*k*_*R*_). Starting with a well defined bimodal distribution (A1) and (B1), for a fixed level of extrinsic noise, the repressed peak can be buffered (A2) or not (B2) depending on the value of *k*_*R*_. If the initial distribution is unimodal repressed (C1), for a given range of parameters, it can be converted into a unimodal unrepressed (C3), crossing a bimodal state (C2), by increasing the protein stability. In (A) the parameters are *k*_*R*_ = 3.1 × 10^−3^ nM min^−1^, ${\bar{k}}_{S}=1.2\times 10^{-3}\;\text{nM}\;{\text{min}}^{-1}$, *σ* = 2.4 × 10^−4^ nM min^−1^, *g*_*S*_ = 1.2 × 10^−2^ min^−1^, *g*_*R*_ = 2.4 × 10^−2^ min^−1^, *g* = 1.2 × 10^2^ nM^−1^ min^−1^, *α* = 0.5, *k*_*P*_ = 6.0 min^−1^, *g*_*P*_ = 2.4 × 10^−2^ min^−1^ for (A1) and *k*_*P*_ = 6.0 × 10^−1^ min^−1^, *g*_*P*_ = 2.4 × 10^−3^ min^−1^ for (A2). In (B) the parameters are *k*_*R*_ = 3.0 × 10^−3^ nM min^−1^, ${\bar{k}}_{S}=1.2\times 10^{-3}\;\text{nM}\;{\text{min}}^{-1}$, *σ* = 2.4 × 10^−4^ nM min^−1^, *g*_*S*_ = 1.2 × 10^−2^ min^−1^, *g*_*R*_ = 2.4 × 10^−2^ min^−1^, *g* = 1.2 × 10^2^ nM^−1^ min^−1^, *α* = 0.5, *k*_*P*_ = 6.0 min^−1^, *g*_*P*_ = 2.4 × 10^−2^ min^−1^ for (B1) and *k*_*P*_ = 6.0 × 10^−1^ min^−1^, *g*_*P*_ = 2.4 × 10^−3^ min^−1^ for (B2). In (C) the parameters are *k*_*R*_ = 3.1 × 10^−3^ nM min^−1^, ${\bar{k}}_{S}=1.4\times 10^{-3}\;\text{nM}\;{\text{min}}^{-1}$, *σ* = 1.7 × 10^−4^ nM min^−1^, *g*_*S*_ = 1.2 × 10^−2^ min^−1^, *g*_*R*_ = 2.4 × 10^−2^ min^−1^, *g* = 1.2 × 10^2^ nM^−1^ min^−1^, *α* = 0.5, *k*_*P*_ = 6.0 min^−1^, *g*_*P*_ = 2.4 × 10^−2^ min^−1^ for (C1), *k*_*P*_ = 3.0min^−1^, *g*_*P*_ = 1.2 × 10^−2^ min^−1^ for (C2) and *k*_*P*_ = 1.2min^−1^, *g*_*P*_ = 4.8 × 10^−3^ min^−1^ for (C3). The ratio between *k*_*P*_ and *g*_*P*_ is always kept constant in order to maintain the mean of the protein distributions at a fixed value. Note that we do not present the analytic curves for such cases as the approximation fails to capture subtle features such as the (dis-)appearance of a small peak (see SI).

Given the relevance of the final product of gene expression, it is important to consider what is the effect of extrinsic noise on proteins’ distributions. From the deterministic system, one can see that the mean amount of target protein is proportional to the amount of its unrepressed mRNA. That is, those molecules not bound to miRNAs and free for translation. In the presence of extrinsic noise the target mRNA can show bimodality even without having a clear double steady state in the deterministic system. Here we investigate if this is the case for the protein distribution. A key factor to keep account of is the time scale of protein synthesis and degradation. In general, if the protein dynamics are fast, the protein distribution follows closely that of the mRNA (see Fig 6A1, 6B1 and 6C1). Conversely, slower protein dynamics tend to filter out the intrinsic fluctuations of the mRNA and lead to narrower distributions (see the SI for a detailed discussion of the case without extrinsic noise). This is a single cell effect. That is, for a given rate of miRNA transcription *k*_*s*_, the corresponding protein distribution gets more peaked as the protein dynamics get slower. The protein distribution subject to extrinsic noise also tends to concentrate around its mean. This feature has different consequences for the protein distribution shape according to the specific structure of the mRNA distribution. If the mRNA distribution is bimodal, slower proteins will have a distribution condensing around their mean, which is located close to the unrepressed peak. They will therefore preferentially display the unrepressed phenotype and may completely lose their bimodal structure (see Fig 6A2). Bimodality can persist for strongly bimodal mRNA distributions because the noise reduction mechanism is acting at the single cell level. Hence it cannot overcome the effects of the extrinsic noise (see Fig 6B1 and 6B2).

For a repressed (unimodal) mRNA distribution the mode is far from the mean, so the narrowing around the mean implies the rise of a second (unrepressed) peak. For moderately slower dynamics (see Fig 6C2) the protein distribution may be bimodal, and for even slower ones it will be unimodal close to its mean (see Fig 6C3).

Altogether these results suggest that slow proteins promote the expression closer to the mean of the corresponding mRNA distribution. This may or may not be sufficient to remove the bimodal feature of the protein distribution depending on the interplay between the amplitude of the extrinsic noise, the coupling between target and miRNA, and the transcription rates.

### Bimodality in endogenous scenarios, time-fluctuating miRNA-production rates and fold repression

**Fig 7:**
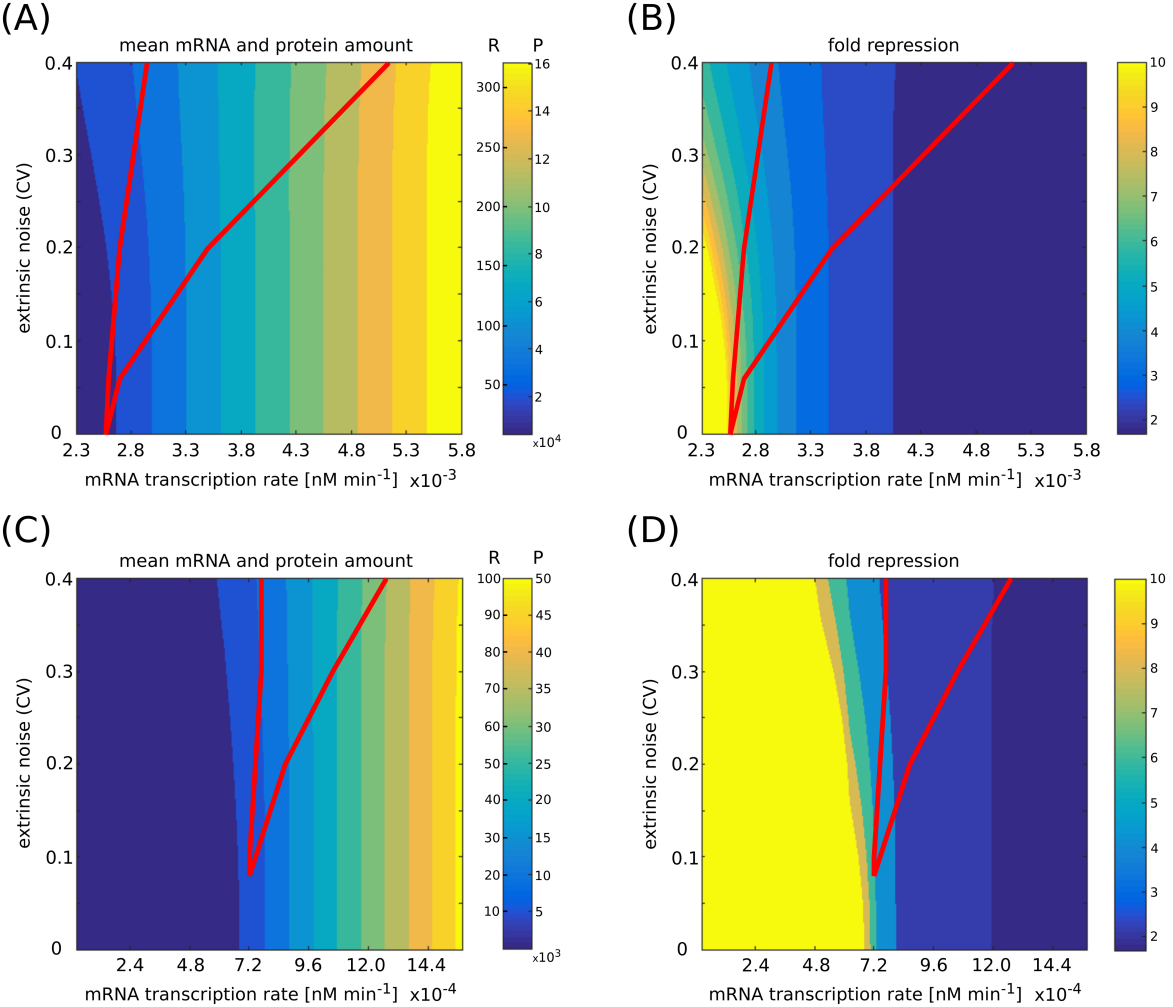
Mean molecules amounts and fold repression. (A,C) Mean mRNA free amount (R) and protein amount (P) for two different sets of parameters, as a function of mRNA transcription rate and extrinsic noise level. The red line indicates the bimodality region. (B,D) Fold repression, i.e. ratio between the unregulated and regulated expression level, as a function of mRNA transcription rate and extrinsic noise level. The red line indicates the bimodality region. Mean values and fold repression are computed through the analytic approximation, while the bimodality region is obtained from numerical simulations. The set of parameters of panels (C) and (D) resembles an endogenous scenario, where the mean values of free mRNAs are of order of tens and the fold repression ranges between 2 and 6. In (A) and (B) the parameters are: *g*_*S*_ = 1.2 × 10^−2^ min^−1^, *g*_*R*_ = 2.4 × 10^−2^ min^−1^, *k*_*P*_ = 6.0 min^−1^, *g*_*P*_ = 1.2 × 10^−2^ min^−1^, *g* = 3.0 × 10^2^ nM^−1^ min^−1^, *α* = 0.5. Target mRNA transcription rate ranges from *k*_*R*_ = 2.3 × 10^−3^ nM min^−1^ to *k*_*R*_ = 5.8 × 10^−3^ nM min^−1^. Extrinsic noise is tuned by varying the standard deviation of the distribution with mean ${\bar{k}}_{S}=1.2\times 10^{-3}\;\text{nM}\;{\text{min}}^{-1}$ from which miRNA transcription rates are picked. The amount of free miRNAs in this regime is of the order of tens, while its total amount (measured as the ratio between its transcription and degradation rates) is 250 molecules per cell. In (C) and (D) the parameters are: *g*_*S*_ = 1.2 × 10^−2^ min^−1^, *g*_*R*_ = 2.4 × 10^−2^ min^−1^, *k*_*P*_ = 6.0 min^−1^, *g*_*P*_ = 1.2 × 10^−2^ min^−1^, *g* = 3.0 × 10^2^ nM^−1^ min^−1^, *α* = 0.8. Target mRNA transcription rate ranges from *k*_*R*_ = 0 nM min^−1^ to *k*_*R*_ = 1.5 × 10^−3^ nM min^−1^. Extrinsic noise is tuned by varying the standard deviation of the distribution with mean ${\bar{k}}_{S}=4.8\times 10^{-4}\;\text{nM}\;{\text{min}}^{-1}$ from which miRNA transcription rates are picked. The amount of free miRNAs in this regime is of the order of tens, while its total amount is 100 molecules per cell.

In the previous sections we considered cases in which the average number of molecules at play is not too low so that the analytic approximation is expected to perform well. Here we investigate how likely the titrative mechanism we analysed is to produce bimodal distributions in regimes of fold repression and mean amount of molecules closer to the endogenous case. We define the fold repression as the ratio between the constitutive expression of the target (i.e. the value the target would have in absence of miRNA regulation, when *g* → 0) and the target itself. As discussed in detail in SI, the fold repression decreases greatly when adding even a little offset in the amount of mRNA molecules (see S6 Fig). Since our model has no offset, the fold repression we measured should be taken as an upper bound to the ones obtainable in experiments. Fig 7 shows the phase diagram of bimodality for two sets of mean miRNA transcription rate *k*_*S*_ and miRNA interaction strength *g* plotted against the mean amount of free mRNAs and proteins (Fig 7A and 7C) and against the fold repression (Fig 7B and 7D). Red lines show the bimodality region. As the figure shows, the bimodality region shrinks and shifts upon decreasing the miRNA transcription rate. The mean values of free mRNAs are of order of hundreds in Fig 7A and of order of tens in Fig 7C (as measured in [58, 59]) and the fold repression ranges between 2 and 10. The amount of free miRNAs in this regime is of the order of tens, while its total amount (measured as the ratio between its transcription and degradation rates) is within 250 molecules per cell. This suggests titration interactions and extrinsic noise may give rise to bimodal distributions also in endogenous regimes.

For the sake of simplicity, analytical and numerical tractability, the analysis presented so far was performed by modelling the extrinsic noise as a gaussian random draw of the miRNA transcription rate. The transcription rate was therefore “fixed” at the beginning of each simulation and not assumed to vary in time. Such approximation is expected to describe adequately the scenario where any variation of the miRNA expression rate takes place on longer time scales than the typical ones in the system. However, this might not be the case for some systems: variation of gene expression may happen on time scales of minutes or hour depending on cells’, and more widely organisms’, needs. One expects that, in the limit of very fast extrinsic fluctuations, their effect will average out and the scenario will reduce to the case of intrinsic noise only where the bimodality region is narrow, located in proximity to the threshold and found only for strong interaction between microRNA and target mRNA, as discussed in [12].

**Fig 8:**
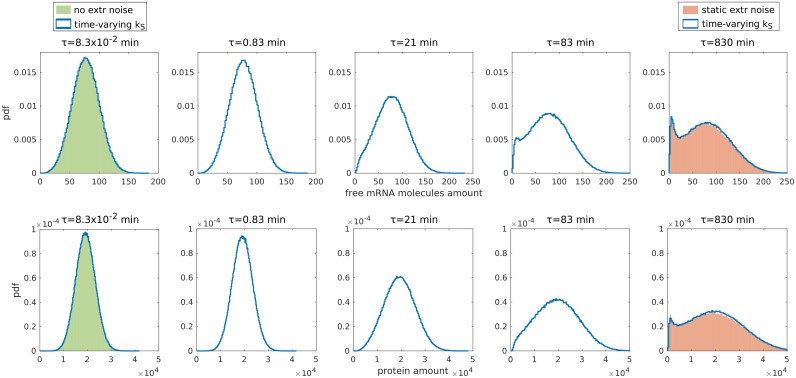
Bimodality appearance in presence of time-dependent extrinsic noise. Solid cyan lines represent free mRNA molecules distributions with a time-dependent extrinsic noise on the miRNA transcription rate *k*_*S*_. The transcription rate is coupled to a birth and death process with finite pool *N* = 100. The steady-state distribution of *k*_*S*_ is a nearly Gaussian distribution with mean ${\bar{k}}_{S}=1.2\times 10^{-3}\;\text{nM}\;{\text{min}}^{-1}$ and standard deviation *σ* = 2.4 × 10^−4^ nM min^−1^. The time scale of this process is tuned by changing the values of the birth and death rates, keeping their ratio fixed. The time scales of the fluctuations of the miRNA transcription rate, from left to right are: 8.3 × 10^−2^ min, 0.83 min, 21 min, 83 min and 830 min. The green histogram in the leftmost plot represents the free mRNA molecules distribution in absence of extrinsic noise. The orange histogram in the rightmost plot represents the free mRNA molecules distribution with static extrinsic noise introduced as described in the main text; the *k*_*S*_ distribution used in this case is a Gaussian with mean ${\bar{k}}_{S}=1.2\times 10^{-3}\;\text{nM}\;{\text{min}}^{-1}$ and standard deviation *σ* = 2.4 × 10^−4^ nM min^−1^. All the free mRNA molecules distributions are obtained from numerical simulations. The parameters are *k*_*R*_ = 3.1 × 10^−3^ nM min^−1^, *g*_*R*_ = 2.4 × 10^−2^ min^−1^, *g*_*S*_ = 1.2 × 10^−2^ min^−1^, *g* = 1.2 × 10^2^ nM^−1^ min^−1^, *α* = 0.5.

To confirm these expectations and to investigate the case in which extrinsic fluctuations take place on time scales comparable to the ones of the other reactions, we performed Gillespie simulations allowing the transcription rate of the microRNA to fluctuate in time. In more detail, we first set all parameters in the system as in Fig 6, where bimodality was observed for static extrinsic noise. We then realised a dynamically fluctuating microRNA transcription rate via an auxiliary birth and death process with finite pool *N* = 100 (see SI). The steady-state distribution of *k*_*S*_ is a Binomial that closely approximates a Gaussian distribution with mean ${\bar{k}}_{S}=1.2\times 10^{-3}\;\text{nM}\;{\text{min}}^{-1}$ and standard deviation $\sigma_{k_{S}}=2.4\times 10^{-4}\text{nM}\;{\text{min}}^{-1}$, i.e. the same distribution from which we drew the rates in the static case discussed in Fig 6. The time scale *τ* on which the microRNA transcription rate fluctuates can be explored by changing the magnitude of the birth and death rates, keeping their ratio fixed. To probe regimes in which these fluctuations are faster, comparable and slower than the typical time scales of the reactions we let *τ* take the values of 8.3 × 10^−2^ min, 0.83 min, 21 min, 83 min and 830 min (see Fig 8). These should be compared with the typical time scales of the various reactions at play in the system. For the target mRNA and protein, the time scale on which significative changes of concentration may occur ranges between about 10 min and 45 min (see the SI for details on how the time scales were estimated). The numerical results confirm that if the miRNA transcription rate varies very rapidly, the effect of the extrinsic noise is averaged out. Indeed, the first panel on the left in Fig 8 shows how the distributions with fast fluctuating miRNA and without extrinsic noise are the same. Considering progressively slower transcription rate variations, the bimodality gradually reappears, and eventually recovers the static case in the slow-variation regime. For *τ* = 830 min the resulting distributions are practically indistinguishable from the static extrinsic noise case treated in the previous sections. This time scale is comparable to (shorter than) 24 hours (1440 min). Then, we expect the results obtained for static noise to be relevant for settings in which the extrinsic noise is caused by variations along the cell cycle, in cells dividing every 24 hours.

## Discussion

Previous studies pointed out the relevance of extrinsic noise in molecular networks in shaping cell decision making and differentiation [9, 10]. Although extrinsic noise influences gene expression and regulation at different levels, the dominating variability across a cell population seems due to population dynamics [30]: even a monoclonal population has cells in different phases of their cell cycle because of growth and divisions. Such intra-population variability may manifest into heterogeneous expression patterns, which eventually develop a bimodal distribution [62, 63]. When bimodal distributions are observed in gene expression levels, these modes often correspond to different physiological states of the system [1–6].

In this work we addressed the question of the role of extrinsic noise in shaping bimodal gene distributions in the context of miRNA-mediated regulation, both with stochastic modelling and simulations.

As observed in vitro [13], in particular stoichiometric conditions miRNAs may induce bimodality in the expression of their target genes simply due to peculiar titrative interactions. In a theoretical system with pure intrinsic noise, such bimodal distributions can be observed in conditions of high miRNA-target interaction strength and for a small range of target transcription rates [12]. The binding and unbinding reactions between miRNA and target in conditions of quasi equimolarity let the target “jump” from the bound to the unbound state, giving rise to bimodal distributions. This bimodality is observed at a single-cell level: every single cell can indeed switch from one state to the other so that, at a given time, part of the population will be “bound” and part “unbound”.

We showed that introducing some extrinsic noise to the miRNA transcription widens the range of target transcription rates for which one observes target bimodality. In this case the bimodal distributions arise at the population level, made of several cells that are heterogeneous with respect to miRNA expression and therefore amount. Hence, the bimodality arises from the superposition of those unimodal distributions describing the single cells, i.e., each of them obtained for a different value of miRNA transcription rate.

Interestingly, in this framework, a high miRNA-target interaction strength is not necessary to obtain a population-induced bimodal distribution. We showed that extrinsic noise and miRNA-target interaction strength act at similar levels with respect to the bimodality. The interaction strength between miRNA and target in our model takes into account the possibility of different numbers of miRNA binding sites on the mRNA target sequence. Since the miRNA repression on a given target is usually small and possibly diluted over multiple targets, our results suggest that some extrinsic noise can compensate for a low interaction strength in order to obtain differentially expressed phenotypes.

Since every single miRNA may have many different targets that in turn compete for the shared pool of miRNAs, a change in the expression level of one of them may alter the expression of the other ones depending on the prioritisation of their interaction strengths [12, 64]. Once sorted, the interaction strengths would indeed provide the specificity of the interaction among a group of miRNAs and a group of targets, as shown in [12]. While from a purely theoretical point of view, there is in principle no limit on the number of genes that can be indirectly regulated by another one (it is just a matter of parameters to tune), of course in physiological conditions the number of genes involved in the crosstalk may be limited and case specific. If one of these targets is bimodally distributed, then the bimodality may be influenced by the expression level of the other miRNA competitors according to their prioritised interaction strengths, and in turn may induce the expression of other targets to become bimodal. We modelled the simplest version of this scenario considering two targets in competition for the same miRNA and showed that cross regulation is possible even in the case of small miRNA-target interaction strengths if some extrinsic noise is present. In particular, different targets may cross-regulate each other’s bimodal distributions and their interplay is pivotal in stabilising the presence of single phenotypes. This suggests that even if the miRNA repression is low and diluted over a network of multiple targets, the noisy environment makes cross-regulation among them possible.

One may then wonder whether the appearance of bimodality is due to unphysiological amount of molecules or it may appear in endogenous situations, with a mean amount of mRNAs in the order of 10 − 1000 [58, 59] and small fold repressions [13, 18]. As shown in Fig 7, the bimodality region may span values of mean mRNA amount in the order of tens and fold repression values ranging between 1 and 10. The mean amount of free miRNAs corresponding to these values is in the order of tens, while its total amount is below 250 molecules per cell. These values suggest that bimodal distributions of the target might appear in endogenous situations.

The importance of miRNAs in increasing protein noise in highly expressed genes was recently suggested in [29]. This result should be read together with the finding that signalling factors and developmental regulators in embryonic stem cells show bimodal expression patterns only in presence of mature miRNAs [62, 63]. As pointed out in [28], as a whole these studies suggest a role of miRNAs in generating gene-expression variability. Consistently with the cell-to-cell miRNA variability observed in [29], they also suggest that variation in miRNA expression may influence the co-variation of factors that are more likely to fluctuate together to trigger transitions between cell states.

The outcome of regulatory systems is the control of protein expression. Concerning the effect of extrinsic noise on their distribution, we showed that depending on the time scales of protein synthesis and degradation, the protein distribution may suppress or amplify the bimodality inherited from the mRNA.

Altogether our results suggest that the coupling between extrinsic noise and threshold behaviour represents a possible mechanism to obtain bimodal phenotypes without the need for fine tuning the rates of reactions which was required for the case of intrinsic noise only. On one hand, we observed that the system is, for a broad range of parameters, able to buffer the extrinsic fluctuations and channel only one final phenotype (unimodality region in Figs 4 and 7). On the other hand, we have highlighted how extrinsic noise widens the bimodality region compared to the intrinsic-noise-only case. This suggests how bimodality can arise as a built-in effect of the coupling between the miRNA-based titration and the presence of noise. This may contribute to the understanding of the bimodal distributions observed in [13]. Such bimodality may play a role in view of the advantages related to having a high variability among different cells.

Given an estimate of the miRNA-target interaction strengths, the model allows the prediction of the amount of extrinsic noise necessary to induce a bimodal phenotype. A feasible strategy to test the experimental validity of these theoretical results involves building ad-hoc synthetic circuits made of miRNA targets tagged with fluorescent labels, as previously done in [13, 65], and performing transfection experiments. Even though the reporters are expressed at artificial levels and with artificial dynamics, with the appropriate reporter-controls the experimental set-up is controlled enough to check the validity of the model. By analysing the fluorescence patterns of the targets interacting with the miRNA throughout the entire population of cells, the shape of the target distributions can then be extracted and compared to the theoretical predictions. MiRNAs are differentially expressed in different tissues and we also expect the amplitudes of their extrinsic fluctuations to vary consequently. Hence, repeating the experiment in cells derived from different tissues would allow the study of cells exposed to different levels of extrinsic noise. This approach would enable the construction of a phase diagram for the bimodality linking extrinsic noise and model parameters, as done in [13]. Nowadays, relevant experiments should use CRISPR-tagged endogenous proteins and inducible systems for miRNA expression to move a physiological system into the desired region of the phase diagram. This would provide a valuable and precise tool to define and control the key variables for the appearance of bimodal target distributions, particularly in disease-related contexts.

## Supporting information

S1 File Details on different approximation methods, supplementary analysis, and simulations. (PDF)

S1 FigComparison between Van Kampen and Gaussian approximations.In (A-C) mRNA and protein distributions for unstable and stable proteins are shown together with the two approximations. In (D) the mean number of mRNA molecules as a function of the miRNA transcription rate is shown, the blue line corresponds to numerical simulations, while the red and black one are the theoretical predictions obtained through the Van Kampen and Gaussian approximation respectively. The inset is a zoom of the region where the difference between the two approximations is more evident. In (A) the parameters are *k*_*R*_ = 3.1 × 10^−3^ nM min^−1^, ${\bar{k}}_{S}=1.2\times 10^{-3}\;\text{nM}\;{\text{min}}^{-1}$, $\sigma_{S}^{2}=2.4\times 10^{-4}\;\text{nM}\;{\text{min}}^{-1}$, *g*_*S*_ = 1.2 × 10^−2^ min^−1^, *g*_*R*_ = 2.4 × 10^−2^ min^−1^, *g* = 1.2 × 10^2^ nM^−1^ min^−1^, *α* = 0.5, *k*_*P*_ = 6.0 min^−1^, *g*_*P*_ = 2.4 × 10^−2^ min^−1^ for (A1) and *k*_*P*_ = 6.0 × 10^−1^ min^−1^, *g*_*P*_ = 2.4 × 10^−3^ min^−1^ for (A2). In (B) the parameters are *k*_*R*_ = 3.0 × 10^−3^ nM min^−1^, ${\bar{k}}_{S}=1.2\times 10^{-3}\;\text{nM}\;{\text{min}}^{-1}$, $\sigma_{S}^{2}=2.4\times 10^{-4}\;\text{nM}\;{\text{min}}^{-1}$, *g*_*S*_ = 1.2 × 10^−2^ min^−1^, *g*_*R*_ = 2.4 × 10^−2^ min^−1^, *g* = 1.2 × 10^2^ nM^−1^ min^−1^, *α* = 0.5, *k*_*P*_ = 6.0 min^−1^, *g*_*P*_ = 2.4 × 10^−2^ min^−1^ for (B1) and *k*_*P*_ = 6.0 × 10^−1^ min^−1^, *g*_*P*_ = 2.4 × 10^−3^ min^−1^ for (B2). In (C) the parameters are *k*_*R*_ = 3.1 × 10^−3^ nM min^−1^, ${\bar{k}}_{S}=1.4\times 10^{-3}\;\text{nM}\;{\text{min}}^{-1}$, $\sigma_{S}^{2}=1.7\times 10^{-4}\;\text{nM}\;{\text{min}}^{-1}$, *g*_*S*_ = 1.2 × 10^−2^ min^−1^, *g*_*R*_ = 2.4 × 10^−2^ min^−1^, *g* = 1.2 × 10^2^ nM^−1^ min^−1^, *α* = 0.5, *k*_*P*_ = 6.0 min^−1^, *g*_*P*_ = 2.4 × 10^−2^ min^−1^ for (C1), *k*_*P*_ = 3.0min^−1^, *g*_*P*_ = 1.2 × 10^−2^ min^−1^ for (C2) and *k*_*P*_ = 1.2min^−1^, *g*_*P*_ = 4.8 × 10^−3^ min^−1^ for (C3). In (D) the parameters are *k*_*R*_ = 3.1 × 10^−3^ nM min^−1^, *g*_*S*_ = 1.2 × 10^−2^ min^−1^, *g*_*R*_ = 2.4 × 10^−2^ min^−1^, *g* = 1.2 × 10^2^ nM^−1^ min^−1^, *α* = 0.5, *k*_*S*_ ranges from 0 to 2.6 × 10^−3^ nM min^−1^.(PDF)

S2 FigComparison between the bimodal mRNA noisy distribution and the weighted superposition of distributions obtained without noise for different miRNA transcription rates.The parameters are the following: *k*_*R*_ = 3.1 × 10^−3^ nM min^−1^, *g*_*S*_ = 1.2 × 10^−2^ min^−1^, *g*_*R*_ = 2.4 × 10^−2^ min^−1^, *g* = 1.2 × 10^2^ nM^−1^ min^−1^, *k*_*P*_ = 6.0 min^−1^, *g*_*P*_ = 1.2 × 10^−2^ min^−1^ and *α* = 0.5. In the main plot the different mRNA distributions correspond, from left to right, to *k*_*S*_ = 1.7 × 10^−3^, 1.4 × 10^−3^, 1.2 × 10^−3^, 9.5 × 10^−4^, 7.1 × 10^−4^ nM min^−1^. In the inset, the mRNA histogram is the result of miRNA transcription rates picked from a gaussian distribution with mean ${\bar{k}}_{S}=1.2\times 10^{-3}\;\text{nM}\;{\text{min}}^{-1}$ and standard deviation *σ* = 2.4 × 10^−4^ nM min^−1^. The black line is the result of the weighted superposition of the distributions represented in the main plot.(PDF)

S3 FigAnalytical prediction for the coefficient of variation.Analytical predictions for the target coefficient of variation in case of one (A) or two (B) targets. In (A) the parameters are ${\bar{k}}_{S}=1.2\times 10^{-3}\;\text{nM}\;{\text{min}}^{-1}$, *σ* = 4.8 × 10^−4^ nM min^−1^, *g*_*S*_ = 1.2 × 10^−2^ min^−1^, *g*_*R*_ = 2.4 × 10^−2^ min^−1^, *g* = 1.2 × 10^2^ nM^−1^ min^−1^, *k*_*P*_ = 6.0 min^−1^, *g*_*P*_ = 1.2 × 10^−2^ min^−1^, *α* = 0.5. *k*_*R*_ ranges from 2.4 × 10^−4^ nM min^−1^ to 5.2 × 10^−3^ nM min^−1^. In (B) the parameters are ${\bar{k}}_{S}=1.2\times 10^{-3}\;\text{nM}\;{\text{min}}^{-1}$, *σ* = 4.8 × 10^−4^ nM min^−1^, *g*_*S*_ = 1.2 × 10^−2^ min^−1^, *g*_*R*1_ = *g*_*R*2_ = 2.4 × 10^−2^ min^−1^, *g*_1_ = 1.2 × 10^2^ nM^−1^ min^−1^, *g*_2_ = 30 nM^−1^ min^−1^, *k*_*P*1_ = *k*_*P*2_ = 6.0 min^−1^, *g*_*P*1_ = *g*_*P*2_ = 1.2 × 10^−2^ min^−1^, *α* = 0.5, *k*_*R*2_ = 9.5 × 10^−4^ nM min^−1^. *k*_*R*1_ ranges from 2.4 × 10^−4^ nM min^−1^ to 5.2 × 10^−3^ nM min^−1^.(PDF)

S4 FigBimodality phase diagram.The plot shows the bimodality phase diagram for the mRNA 1 in a system with two targets competing for the same miRNA. The parameters here used are the following: ${\bar{k}}_{S}=1.2\times 10^{-3}$ nM min^−1^, *σ*_*S*_ = 2.4 × 10^−4^ nM min^−1^, *g*_1_ = 1.2 × 10^2^ nM^−1^ min^−1^, *k*_*R*1_ and *k*_*R*2_ range from 0 nM min^−1^ to 5.1 × 10^−3^ nM min^−1^, *g*_*S*_ = 1.2 × 10^−2^ min^−1^, *g*_*R*1_ = *g*_*R*2_ = 2.4 × 10^−2^ min^−1^, *k*_*P*1_ = *k*_*P*2_ = 6.0 min^−1^, *g*_*P*1_ = *g*_*P*2_ = 2.4 × 10^−2^ min^−1^, *α* = 0.5.(PDF)

S5 FigBimodality amplitude phase diagram.Phase diagram of the bimodality amplitude of the mRNA distribution as a function of the mRNA transcription rate *k*_*R*_ and of the extrinsic noise level. The parameters here used are the following: *g*_*S*_ = 1.2 × 10^−2^ min^−1^, *g*_*R*_ = 2.4 × 10^−2^ min^−1^, *g* = 3.0 × 10^2^ nM^−1^ min^−1^, *k*_*P*_ = 6.0 min^−1^, *g*_*P*_ = 1.2 × 10^−2^ min^−1^, *α* = 0.5. Target mRNA transcription rate is one of the control parameters and ranges from *k*_*R*_ = 2.6 × 10^−3^ nM min^−1^ to *k*_*R*_ = 5.1 × 10^−3^ nM min^−1^. Extrinsic noise is tuned by varying the standard deviation of the distribution with mean ${\bar{k}}_{S}=1.2\times 10^{-3}\;\text{nM}\;{\text{min}}^{-1}$ from which miRNA transcription rates are picked. The standard deviation ranges from *σ* = 0 nM min^−1^ (no extrinsic noise) to *σ* = 3.6 × 10^−4^ nM min^−1^. This phase diagram was obtained by interpolating the single distributions obtained from numerical simulations as described in Sec. V. The green line represents the separation between bimodal and unimodal regions as shown in the Main Text.(PDF)

S6 Fig The role of the offset. (A) Example of two average mRNA profiles, for a regulated (orange) and an unregulated (blue) mRNA. (B) Same profile as panel A but with the curves shifted upwards by an arbitrary offset of 10 mRNA molecules. (C) Fold repression (ratio of blue to orange curve from panel A) without the offset. (D) Fold repression (ratio of blue to orange curve from panel B) with the offset. (E) Comparison of the plots of fold repression with offset (from panel D) and without offset (from panel C). The parameters here used are the following: *g*_*S*_ = 1.2 × 10^−2^ min^−1^, *g*_*R*_ = 2.4 × 10^−2^ min^−1^, *g* = 3.0 × 10^1^ nM^−1^ min^−1^, *k*_*S*_ = 7.1 × 10^−4^ nM min^−1^, *α* = 0.5. *k*_*R*_ ranges from 0 nM min^−1^ to 4.8 × 10^−3^ nM min^−1^.
(PDF)
