# Supplementary material for: On the role of extrinsic noise in microRNA-mediated bimodal gene expression
Source: PLoS Comput Biol. 2018 Apr 17;14(4):e1006063. doi: 10.1371/journal.pcbi.1006063 (PMC5922620; doi:10.1371/journal.pcbi.1006063)
Supplement: S1 File — (PDF) [file pcbi.1006063.s001.pdf]

# On the role of extrinsic noise in microRNA-mediated bimodal gene expression - Supplementary Information

Marco Del Giudice, Stefano Bo, Silvia Grigolon, Carla Bosia

## I. COMPARISON BETWEEN DIFFERENT ANALYTICAL APPROXIMATIONS

In the Main Text we showed how to obtain the steady state distribution $P(n_S, n_R, n_P|k_S)$ conditioned on a specific miRNA transcription rate by a system-size expansion [1]. Alternatively, one could resort to a gaussian approximation of the moments as in [2] which consists in closing the hierarchy of the equations for the moments by expressing the third one as a combination of means and covariances. The simplest way to actually produce the shape of the distribution $P(n_S, n_R, n_P|k_S)$ is to make the additional assumption that it is gaussian with averages and covariances given by the moment closure scheme. Both approximations can be convoluted with the distribution of $k_S$ to produce the complete distribution $P(n_S, n_R, n_P)$.

In this section we compare the results of the two approximations. For the plots in Figure 2 and 4 in the Main Text the two approximations give rise to basically indistinguishable curves (not shown) in agreement with the simulation results. However, they may fail to predict subtle details in the bimodal distribution, such as the initial emergence of the unrepressed peak addressed in Fig. 5 in the Main Text. For such parameters, the two approximations differ more markedly (see Fig. S1). In general, the gaussian approximation is more accurate in predicting the shape of the protein distribution in case of high protein stability. This observation can be traced back to the fact that the gaussian approximation over-performs the van Kampen one in describing the mean value of mRNA and proteins (see Fig. S1 D). As discussed in the Main Text, proteins with slower dynamics narrow their distribution around their mean so that predicting the mean accurately becomes more relevant. In Figure S1 we show a comparison between the two approximations in relation to the histograms represented in Fig. 5 in the main text.

However, when the timescales of mRNAs and proteins are comparable there seems to be no general rules on when one approximation is to be preferred. For the investigation of subtle features of the distributions, the approximation should be just used as a qualitative reference to be validated by simulations.

## II. THE COEFFICIENT OF VARIATION AS AN INDICATOR OF BIMODALITY LOSS IN CASE OF PURE INTRINSIC NOISE

The comparison of coefficient of variations ($CV$) are common measures of noise buffering in molecular networks. Here we focus on the comparison between the coefficient of variations of the targets ($CV_R$) and the proteins ($CV_P$) in case of pure intrinsic noise. If there is noise buffering, i.e. the amount of noise is reduced by the production/degradation of proteins, the protein CV will be smaller than the target one. Conversely, if there is no noise buffering, the two CVs are exactly the same. To give a prediction on the values of the parameters for which noise buffering is present, one can use van Kampen's system-size expansion's solution and build up analytical expressions for the coefficient of variations of targets and proteins. Even by fixing all parameters but miRNA transcription rates and proteins transcription and degradation rates, the expression for the coefficient of variations are quite cumbersome. To understand qualitatevely the behaviour of the Coefficient of Variation as a function of the degradation rate of the proteins, one can fix all the other parameters and compute the expression of the CV. Imposing $CV_P - CV_R \leq 0$ allows to quantify the range of values of $g_P$ to have noise buffering in the final protein channel. In particular we found that $g_P \leq 0.9$ min$^{-1}$ for the parameters' choice as in Fig. 5A-A1, i.e., for values of $g_P$ smaller than a given threshold and hence for long-living proteins.

## III. BIMODAL DISTRIBUTIONS IN THE LOW-MOLECULES REGIME

In the Main Text, we deeply analysed the appearance of targets' and proteins' bimodal distributions for numbers of molecules of order hundreds or thousands. However, physiologically, one may often encounter a very low number of expressed targets, and consequently proteins (as reported for different protein families on Bionumbers [3]). In this low-molecules regime, the difference between a repressed and expressed state might lose its meaning along with the appearance of bimodality. To test whether this is the case, we first reduced the average number of targets and miRNA, therefore shifting the position of the threshold in the target-expression diagram plotted in Figure 3A. Although the number of molecules is low, the target average is well-captured by the van Kampen approximation. We then proceeded as explained in the Main Text by running Gillespie's simulations for different target tran-

scription rates and sampling both targets' and proteins' distributions in presence of extrinsic noise. By increasing the target transcription rate, $k_r$, the distributions show different shapes as discussed in the Main Text. For low $k_r$ the distributions appear very peaked around low values of targets and proteins. For intermediate $k_r$, bimodal distributions appear again, underlining that this property is peculiar to this system and driven by the nature of noise, independent of the numbers' order of magnitude. Finally, for high $k_r$, the repressed peak disappears and the distributions turn back to be unimodal (Fig. 3B,C in the Main Text). The main difference with the situation described in Figure 2 of the Main Text is in the agreement with the van Kampen approximation: the height of the peaks is not well-captured by the analytical distribution. This result is somehow expected as the van Kampen approximation is exact for infinite molecules' number. However, surprisingly, it still captures the shape of the distribution in all the different scenarios pictured in Figure 3B,C in the Main Text.

## IV. BIMODALITY AMPLITUDE AS AN INDICATOR OF BIMODALITY APPEARANCE

Throughout the entire work, we were interested in what mechanisms in the model could induce the appearance or not of bimodal targets' distributions. However, how probability distributions change shape during the unimodal-bimodal transition is also an interesting point. To perform this analysis, we computed the bimodality amplitude [4]:

$$A_B = \frac{A_1 - A_{an}}{A_1} \tag{1}$$

where $A_1$ is the amplitude of the smallest peak, while $A_{an}$ is amplitude of the local minimum between the two peaks. The bimodality amplitude is by definition smaller than 1: the higher the values, the more separated the maxima. To study how this coefficient varies as a function of the extrinsic noise in the system and the target transcription rate, we first sampled several different targets' probability distributions as in Fig. 3A. By interpolating these distributions with built-in functions in Matlab, we extracted the positions of the maxima and minima as well as their frequency which allowed us to compute the bimodality amplitude. The resulting phase diagram is shown is Fig S7. Bimodal distributions appear for a very well-defined range of parameters in agreement with what found in Fig. 3A. The transition from unimodal to bimodal appears to be peaked around some particular values of

the noise coefficient of variation and the target transcription rate, suggesting the existence of optimal values for these parameters. However, it would be even more interesting to study in the future how smoothly bimodal distributions appear in this parameters' space and whether this observed peak could really be an optimal point for cell variability.

## V. FOLD CHANGE MEASUREMENTS

In the experimental framework [5, 6], fold repression is measured as the ratio between the unregulated and the regulated level of expression of the miRNA target. As it can be observed in Fig. S6, the fold-repression profile is significantly sensitive to offsets in the data. Indeed, if the offset is zero or close to zero (panel (A)), for low values of constitutive expression, the fold repression, i.e. the ratio between the blue and the orange line in the plot, is high and then monotonically decreases (panel (C)). With an initial offset (panel (B)), the fold repression is equal to 1 in the beginning, then shows a maximum and finally tends to a constant value (panel (D)). The two profiles, then, differ strongly, as shown in the comparison given in panel (E). The mechanism generating the profile is the same but the fold repression is an observable strongly affected by the free mRNA offset, which in fluorescence reporter experiments, is not a clearly controlled quantity. Since our model has no offset, the fold repression we measured should be taken as an upper bound to the ones normally obtained in experiments.

## VI. TIME SCALES AND BIMODALITY APPEARANCE IN PRESENCE OF EXTRINSIC-NOISE TEMPORAL FLUCTUATIONS

We first consider the constraints set by cell division on the degradation rates of the system. If we assume a doubling time of 24 hours, this implies a typical corresponding degradation rate of the order of $ln(2)/(24*60) \sim 4.8 \times 10^{-4}$ min$^{-1}$. The degradation rates we have used in the case studies, as reported in the legend of the figures, vary between $1.2 \times 10^{-2}$ min$^{-1}$ and $2.4 \times 10^{-2}$ min$^{-1}$ for most cases and, when investigating slow protein dynamics, are pushed to $2.4 \times 10^{-3}$ min$^{-1}$. These degradations are at least one order of magnitude faster then the lower bound set by cell division, which is then, clearly, not a concern for our results.

In order to investigate the effects of extrinsic-noise temporal fluctuations, we first need

to evaluate the typical time scales of the reactions involved in the system. One can estimate these time scales by looking at the rate equation (5) and inserting the steady state concentrations. The rate at which one observes an appreciable change in a concentration is then obtained by dividing the propensities by the concentrations and the typical time scale is just its inverse. For the chosen parameters, the solution of the rate equation gives an mRNA concentration of $[R] = 0.03$ nM and a miRNA concentration of $[S] = 6.6 \times 10^{-4}$ nM. Note that these are just indicative values and differ slightly from the averages stemming from the numerical simulations with extrinsic noise. For the case of the miRNA-target interaction, we evaluated the time scales related to a change of concentration of both the miRNA and the mRNA. The values of the time scales are reported in Table I.

To study the dynamic noise case, we performed Gillespie simulations allowing the transcription rate of the microRNA to fluctuate in time. In practice, we first set all parameters in the system as in Figure 6A, where bimodality was observed for static extrinsic noise. We then realised a dynamically fluctuating microRNA transcription rate via a birth and death process with finite pool $N = 100$. This is done via the auxiliary variable X that obeys the master equation:

$$\frac{dP(X)}{dt} = k_X(N-X+1)P(X-1) + g_X(X+1)P(X+1) - [k_X(N-X) + g_X X]P(X), \quad (2)$$

which has a binomial steady-state distribution with average 20 and standard deviation 4, closely resembling to a Gausssian. The rate $k_S$ is obtained by multiplying $X * 6 \times 10^{-5}$ nM min$^{-1}$ and has a steady-state distribution that closely approximates a Gaussian distribution with mean $\langle k_S \rangle = 1.2 \times 10^{-3}$ nM min$^{-1}$ and standard deviation $\sigma_{k_S} = 2.4 \times 10^{-4}$ nM min$^{-1}$, i.e. the same distribution from which we drew the rates in the static case discussed in Figure 6. As discussed for the other reactions, the time scales of the extrinsic fluctuations can be estimated by dividing the average amount of the auxiliary species by the average propensities of birth and death at the steady state. This identifies a typical time scale on which the abundance of the auxiliary species X varies appreciably $\tau = \frac{1}{g_X} = \frac{<X>}{(N-<X>)k_x}$. Keeping the ratio $k_X/g_X = 0.25$ nM fixed and varying $k_X$ and $g_X$ we can explore different time scales ($\tau$) of the fluctuations of the microRNA transcription rates. To probe regimes in which these fluctuations are faster, comparable and slower than the typical time scales of the reactions we let $\tau$ take values of $8.3 \times 10^{-2}$ min, 0.83 min, 21 min, 83 min and 830 min.

| reaction | time scale (min) |
|---|---|
| $k_R$ | 10 |
| $g_R$ | 42 |
| $k_S$ | 0.5 |
| $g_S$ | 83 |
| $g$ (mRNA) | 13 |
| $g$ (miRNA) | 0.3 |
| $k_P$ | 42 |
| $g_P$ | 42 |
| $k_X$ | $8.3 \times 10^{-2}$ – 830 |
| $g_X$ | $8.3 \times 10^{-2}$ – 830 |
| cell division | 1440 |

**TABLE I:** Time scales of all reactions involved in the network. Each reaction is identified by its corresponding rate.

These should be compared with the typical time scales of the various reactions at play in the system that are presented in Table I. For the slow dynamic noise with $\tau = 830$ min the resulting distributions are practically indistinguishable from the static extrinsic noise case treated in the previous sections. This time scale is comparable to (shorter) than 24 hours (1440 min). Then, we expect the results obtained for static noise to be relevant for settings in which the extrinsic noise is caused by variations along the cell cycle, in cells dividing every 24 hours.

---


[1] N. G. van Kampen, *Stochastic processes in physics and chemistry.* North-Holland Personal Library, 1981.

[2] C. Bosia, A. Pagnani, and R. Zecchina, "Modelling competing endogenous rna networks," *PLoS One*, vol. 8(6), p. e66609, 2013.

[3] R. Milo, P. Jorgensen, U. Moran, G. Weber, and M. Springer, "BioNumbers—the database of key numbers in molecular and cell biology," *Nucleic Acids Research*, vol. 38, pp. D750–3, Jan. 2010.

[4] C. Zhang, B. E. Mapes, and B. J. Soden, “Bimodality in tropical water vapour,” *Quarterly Journal of the Royal Meteorological Society*, vol. 129, no. 594, pp. 2847–2866, 2003.

[5] S. Mukherji, M. Ebert, G. Zheng, J. Tsang, P. Sharp, and A. van Oudenaarden, “Micrornas can generate thresholds in target gene expression,” *Nat Genet*, vol. 43(9), pp. 854–859, 2011.

[6] C. Bosia, F. Sgró, L. Conti, C. Baldassi, D. Brusa, F. Cavallo, F. Di Cunto, E. Turco, A. Pagnani, and R. Zecchina, “Rnas competing for micrornas mutually influence their fluctuations in a highly non-linear microrna-dependent manner in single cells,” *Genome Biology*, vol. 18, p. 37, 2017.
