# Supplementary material for: On the role of extrinsic noise in microRNA-mediated bimodal gene expression
Source: PLoS Comput Biol. 2018 Apr 17;14(4):e1006063. doi: 10.1371/journal.pcbi.1006063 (PMC5922620; doi:10.1371/journal.pcbi.1006063)
Supplement: S1 Fig — In (A-C) mRNA and protein distributions for unstable and stable proteins are shown together with the two approximations. In (D) the mean number of mRNA molecules as a function of the miRNA transcription rate is shown, the blue line corresponds to numerical simulations, while the red and black one are the theoretical predictions obtained through the Van Kampen and Gaussian approximation respectively. The inset is a zoom of the region where the difference between the two approximations is more evident. In (A) the parameters are kR = 3.1 × 10^−3^ nM min−1, k¯S=1.2×10^-3^nMmin-1, σS2=2.4×10^-4^nMmin-1, gS = 1.2 × 10−2 min−1, gR = 2.4 × 10−2 min−1, g = 1.2 × 10^2^ nM−1 min−1, α = 0.5, kP = 6.0 min−1, gP = 2.4 × 10−2 min−1 for (A1) and kP = 6.0 × 10−1 min−1, gP = 2.4 × 10−3 min−1 for (A2). In (B) the parameters are kR = 3.0 × 10^−3^ nM min−1, k¯S=1.2×10^-3^nMmin-1, σS2=2.4×10^-4^nMmin-1, gS = 1.2 × 10−2 min−1, gR = 2.4 × 10−2 min−1, g = 1.2 × 10^2^ nM−1 min−1, α = 0.5, kP = 6.0 min−1, gP = 2.4 × 10−2 min−1 for (B1) and kP = 6.0 × 10−1 min−1, gP = 2.4 × 10−3 min−1 for (B2). In (C) the parameters are kR = 3.1 × 10^−3^ nM min−1, k¯S=1.4×10^-3^nMmin-1, σS2=1.7×10^-4^nMmin-1, gS = 1.2 × 10−2 min−1, gR = 2.4 × 10−2 min−1, g = 1.2 × 10^2^ nM−1 min−1, α = 0.5, kP = 6.0 min−1, gP = 2.4 × 10−2 min−1 for (C1), kP = 3.0min−1, gP = 1.2 × 10−2 min−1 for (C2) and kP = 1.2min−1, gP = 4.8 × 10−3 min−1 for (C3). In (D) the parameters are kR = 3.1 × 10^−3^ nM min−1, gS = 1.2 × 10−2 min−1, gR = 2.4 × 10−2 min−1, g = 1.2 × 10^2^ nM−1 min−1, α = 0.5, kS ranges from 0 to 2.6 × 10^−3^ nM min−1. (PDF) [file pcbi.1006063.s002.pdf]

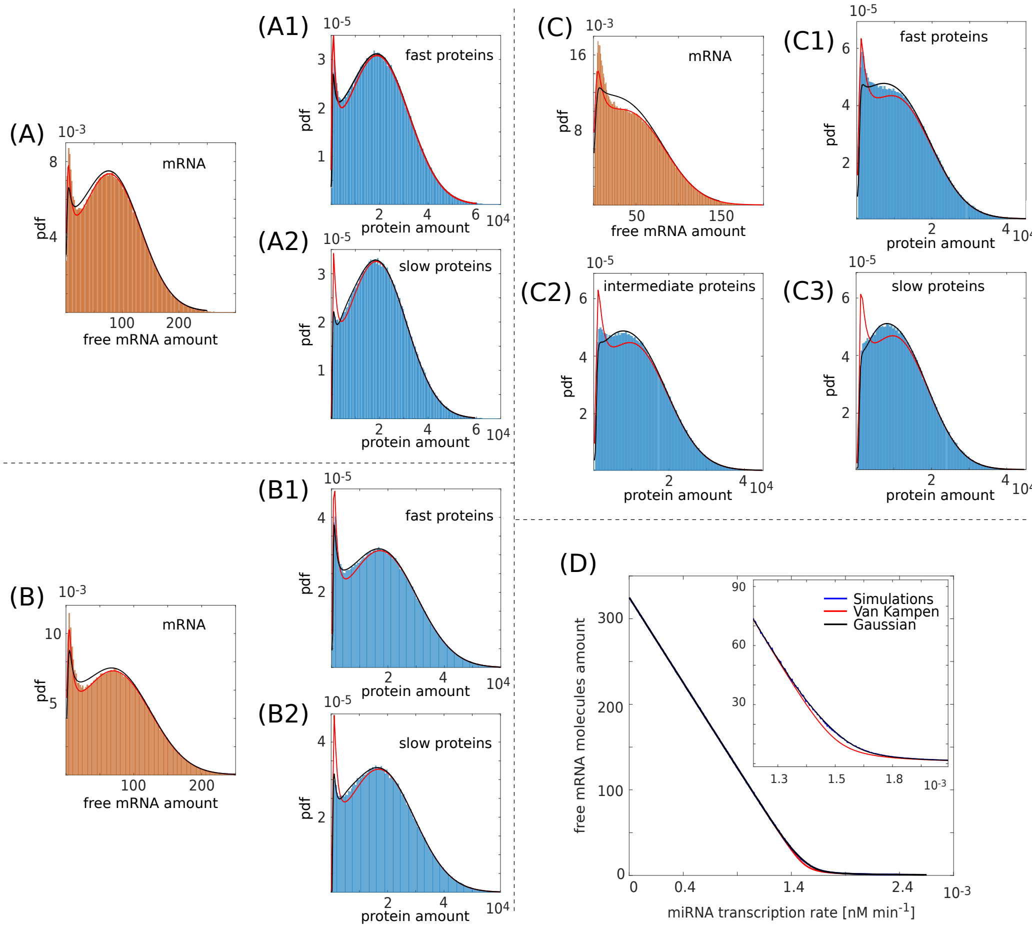

FIG. S1: Comparison between Van Kampen and gaussian approximations. In (A-C) mRNA and protein distributions for unstable and stable proteins are shown together with the two approximations. In (D) the mean number of mRNA molecules as a function of the miRNA transcription rate is shown, the blue line corresponds to numerical simulations, while the red and black one are the theoretical predictions obtained through the Van Kampen and Gaussian approximation respectively. The inset is a zoom of the region where the difference between the two approximations is more evident. In (A) the parameters are $k_R = 3.1 \times 10^{-3}$ nM min^{-1}, $\overline{k}_S = 1.2 \times 10^{-3}$ nM min^{-1}, $\sigma_S^2 = 2.4 \times 10^{-4}$ nM min^{-1}, $g_S = 1.2 \times 10^{-2}$ min^{-1}, $g_R = 2.4 \times 10^{-2}$ min^{-1}, $g = 1.2 \times 10^{2}$ nM^{-1} min^{-1}, $\alpha = 0.5$, $k_P = 6.0$ min^{-1}, $g_P = 2.4 \times 10^{-2}$ min^{-1} for (A1) and $k_P = 6.0 \times 10^{-1}$ min^{-1}, $g_P = 2.4 \times 10^{-3}$ min^{-1} for (A2). In (B) the parameters are $k_R = 3.0 \times 10^{-3}$ nM min^{-1}, $\overline{k}_S = 1.2 \times 10^{-3}$ nM min^{-1}, $\sigma_S^2 = 2.4 \times 10^{-4}$ nM min^{-1}, $g_S = 1.2 \times 10^{-2}$ min^{-1}, $g_R = 2.4 \times 10^{-2}$ min^{-1}, $g = 1.2 \times 10^{2}$ nM^{-1} min^{-1}, $\alpha = 0.5$, $k_P = 6.0$ min^{-1}, $g_P = 2.4 \times 10^{-2}$ min^{-1} for (B1) and $k_P = 6.0 \times 10^{-1}$ min^{-1}, $g_P = 2.4 \times 10^{-3}$ min^{-1} for (B2). In (C) the parameters are $k_R = 3.1 \times 10^{-3}$ nM min^{-1}, $\overline{k}_S = 1.4 \times 10^{-3}$ nM min^{-1}, $\sigma_S^2 = 1.7 \times 10^{-4}$ nM min^{-1}, $g_S = 1.2 \times 10^{-2}$ min^{-1}, $g_R = 2.4 \times 10^{-2}$ min^{-1}, $g = 1.2 \times 10^{2}$ nM^{-1} min^{-1}, $\alpha = 0.5$, $k_P = 6.0$ min^{-1}, $g_P = 2.4 \times 10^{-2}$ min^{-1} for (C1), $k_P = 3.0$ min^{-1}, $g_P = 1.2 \times 10^{-2}$ min^{-1} for (C2) and $k_P = 1.2$ min^{-1}, $g_P = 4.8 \times 10^{-3}$ min^{-1} for (C3). In (D) the parameters are $k_R = 3.1 \times 10^{-3}$ nM min^{-1}, $g_S = 1.2 \times 10^{-2}$ min^{-1}, $g_R = 2.4 \times 10^{-2}$ min^{-1}, $g = 1.2 \times 10^{2}$ nM^{-1} min^{-1}, $\alpha = 0.5$, k_S ranges from 0 to 2.6×10^{-3} nM min^{-1}.
