# Supplementary material for: On the role of extrinsic noise in microRNA-mediated bimodal gene expression
Source: PLoS Comput Biol. 2018 Apr 17;14(4):e1006063. doi: 10.1371/journal.pcbi.1006063 (PMC5922620; doi:10.1371/journal.pcbi.1006063)
Supplement: S2 Fig — The parameters are the following: kR = 3.1 × 10^−3^ nM min−1, gS = 1.2 × 10−2 min−1, gR = 2.4 × 10−2 min−1, g = 1.2 × 10^2^ nM−1 min−1, kP = 6.0 min−1, gP = 1.2 × 10−2 min−1 and α = 0.5. In the main plot the different mRNA distributions correspond, from left to right, to kS = 1.7 × 10^−3^, 1.4 × 10^−3^, 1.2 × 10^−3^, 9.5 × 10^−4^, 7.1 × 10^−4^ nM min−1. In the inset, the mRNA histogram is the result of miRNA transcription rates picked from a gaussian distribution with mean k¯S=1.2×10^-3^nMmin-1 and standard deviation σ = 2.4 × 10^−4^ nM min−1. The black line is the result of the weighted superposition of the distributions represented in the main plot. (PDF) [file pcbi.1006063.s003.pdf]

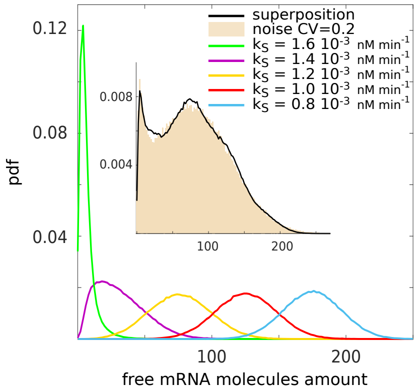

FIG. S2: Comparison between the bimodal mRNA noisy distribution and the weighted superposition of distributions obtained without noise for different miRNA transcription rates. The parameters are the following: $k_R = 3.1 \times 10^{-3}$ nM min^{-1}, $g_S = 1.2 \times 10^{-2}$ min^{-1}, $g_R = 2.4 \times 10^{-2}$ min^{-1}, $g = 1.2 \times 10^2$ nM^{-1} min^{-1}, $k_P = 6.0$ min^{-1}, $g_P = 1.2 \times 10^{-2}$ min^{-1} and $\alpha = 0.5$. In the main plot the different mRNA distributions correspond, from left to right, to $k_S = 1.7 \times 10^{-3}, 1.4 \times 10^{-3}, 1.2 \times 10^{-3}, 9.5 \times 10^{-4}, 7.1 \times 10^{-4}$ nM min^{-1}. In the inset, the mRNA histogram is the result of miRNA transcription rates picked from a gaussian distribution with mean $\overline{k}_S = 1.2 \times 10^{-3}$ nM min^{-1} and standard deviation $\sigma = 2.4 \times 10^{-4}$ nM min^{-1}. The black line is the result of the weighted superposition of the distributions represented in the main plot.
