# Supplementary material for: On the role of extrinsic noise in microRNA-mediated bimodal gene expression
Source: PLoS Comput Biol. 2018 Apr 17;14(4):e1006063. doi: 10.1371/journal.pcbi.1006063 (PMC5922620; doi:10.1371/journal.pcbi.1006063)
Supplement: S3 Fig — Analytical predictions for the target coefficient of variation in case of one (A) or two (B) targets. In (A) the parameters are k¯S=1.2×10^-3^nMmin-1, σ = 4.8 × 10^−4^ nM min−1, gS = 1.2 × 10−2 min−1, gR = 2.4 × 10−2 min−1, g = 1.2 × 10^2^ nM−1 min−1, kP = 6.0 min−1, gP = 1.2 × 10−2 min−1, α = 0.5. kR ranges from 2.4 × 10^−4^ nM min−1 to 5.2 × 10^−3^ nM min−1. In (B) the parameters are k¯S=1.2×10^-3^nMmin-1, σ = 4.8 × 10^−4^ nM min−1, gS = 1.2 × 10−2 min−1, gR1 = gR2 = 2.4 × 10−2 min−1, g1 = 1.2 × 10^2^ nM−1 min−1, g2 = 30 nM−1 min−1, kP1 = kP2 = 6.0 min−1, gP1 = gP2 = 1.2 × 10−2 min−1, α = 0.5, kR2 = 9.5 × 10^−4^ nM min−1. kR1 ranges from 2.4 × 10^−4^ nM min−1 to 5.2 × 10^−3^ nM min−1. (PDF) [file pcbi.1006063.s004.pdf]

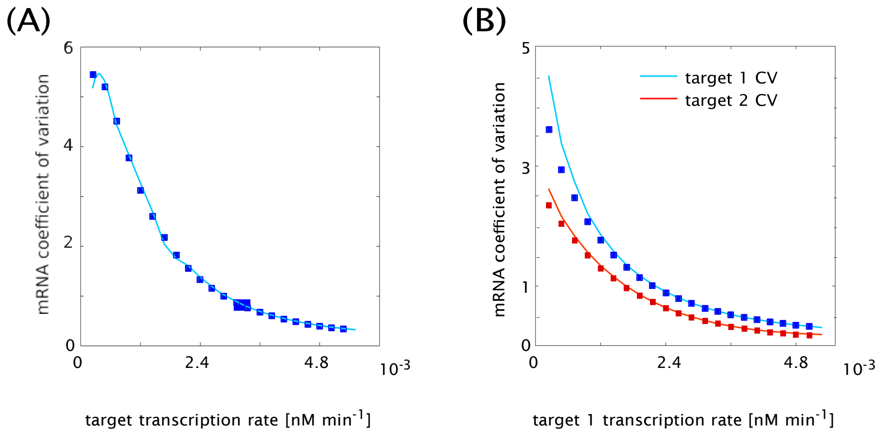

FIG. S3: Analytical prediction for the coefficient of variation. Analytical predictions for the target coefficient of variation in case of one (A) or two (B) targets. In (A) the parameters are $\bar{k_S} = 1.2 \times 10^{-3}$ nM min^{-1}, $\sigma = 4.8 \times 10^{-4}$ nM min^{-1}, $g_S = 1.2 \times 10^{-2}$ min^{-1}, $g_R = 2.4 \times 10^{-2}$ min^{-1}, $g = 1.2 \times 10^{2}$ nM^{-1} min^{-1}, $k_P = 6.0$ min^{-1}, $g_P = 1.2 \times 10^{-2}$ min^{-1}, $\alpha = 0.5$. k_R ranges from 2.4×10^{-4} nM min^{-1} to 5.2×10^{-3} nM min^{-1}. In (B) the parameters are $\bar{k_S} = 1.2 \times 10^{-3}$ nM min^{-1}, $\sigma = 4.8 \times 10^{-4}$ nM min^{-1}, $g_S = 1.2 \times 10^{-2}$ min^{-1}, $g_{R1} = g_{R2} = 2.4 \times 10^{-2}$ min^{-1}, $g_1 = 1.2 \times 10^{2}$ nM^{-1} min^{-1}, $g_2 = 30$ nM^{-1} min^{-1}, $k_{P1} = k_{P2} = 6.0$ min^{-1}, $g_{P1} = g_{P2} = 1.2 \times 10^{-2}$ min^{-1}, $\alpha = 0.5$, $k_{R2} = 9.5 \times 10^{-4}$ nM min^{-1}. k_{R1} ranges from 2.4×10^{-4} nM min^{-1} to 5.2×10^{-3} nM min^{-1}.
