# Supplementary material for: On the role of extrinsic noise in microRNA-mediated bimodal gene expression
Source: PLoS Comput Biol. 2018 Apr 17;14(4):e1006063. doi: 10.1371/journal.pcbi.1006063 (PMC5922620; doi:10.1371/journal.pcbi.1006063)
Supplement: S4 Fig — The plot shows the bimodality phase diagram for the mRNA 1 in a system with two targets competing for the same miRNA. The parameters here used are the following: k¯S=1.2×10^-3^ nM min−1, σS = 2.4 × 10^−4^ nM min−1, g1 = 1.2 × 10^2^ nM−1 min−1, kR1 and kR2 range from 0 nM min−1 to 5.1 × 10^−3^ nM min−1, gS = 1.2 × 10−2 min−1, gR1 = gR2 = 2.4 × 10−2 min−1, kP1 = kP2 = 6.0 min−1, gP1 = gP2 = 2.4 × 10−2 min−1, α = 0.5. (PDF) [file pcbi.1006063.s005.pdf]

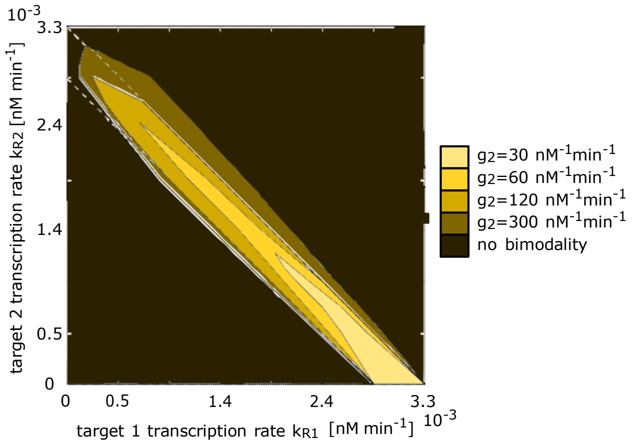

FIG. S4: Bimodality phase diagram. The plot shows the bimodality phase diagram for the mRNA 1 in a system with two targets competing for the same miRNA. The parameters here used are the following: $\bar{k}_S = 1.2 \times 10^{-3}$ nM min^{-1}, $\sigma_S = 2.4 \times 10^{-4}$ nM min^{-1}, $g_1 = 1.2 \times 10^2$ nM^{-1} min^{-1}, k_{R1} and k_{R2} range from 0 nM min^{-1} to 5.1×10^{-3} nM min^{-1}, $g_S = 1.2 \times 10^{-2}$ min^{-1}, $g_{R1} = g_{R2} = 2.4 \times 10^{-2}$ min^{-1}, $k_{P1} = k_{P2} = 6.0$ min^{-1}, $g_{P1} = g_{P2} = 2.4 \times 10^{-2}$ min^{-1}, $\alpha = 0.5$.
