# Supplementary material for: On the role of extrinsic noise in microRNA-mediated bimodal gene expression
Source: PLoS Comput Biol. 2018 Apr 17;14(4):e1006063. doi: 10.1371/journal.pcbi.1006063 (PMC5922620; doi:10.1371/journal.pcbi.1006063)
Supplement: S5 Fig — Phase diagram of the bimodality amplitude of the mRNA distribution as a function of the mRNA transcription rate kR and of the extrinsic noise level. The parameters here used are the following: gS = 1.2 × 10−2 min−1, gR = 2.4 × 10−2 min−1, g = 3.0 × 10^2^ nM−1 min−1, kP = 6.0 min−1, gP = 1.2 × 10−2 min−1, α = 0.5. Target mRNA transcription rate is one of the control parameters and ranges from kR = 2.6 × 10^−3^ nM min−1 to kR = 5.1 × 10^−3^ nM min−1. Extrinsic noise is tuned by varying the standard deviation of the distribution with mean k¯S=1.2×10^-3^nMmin-1 from which miRNA transcription rates are picked. The standard deviation ranges from σ = 0 nM min−1 (no extrinsic noise) to σ = 3.6 × 10^−4^ nM min−1. This phase diagram was obtained by interpolating the single distributions obtained from numerical simulations as described in Sec. V. The green line represents the separation between bimodal and unimodal regions as shown in the Main Text. (PDF) [file pcbi.1006063.s006.pdf]

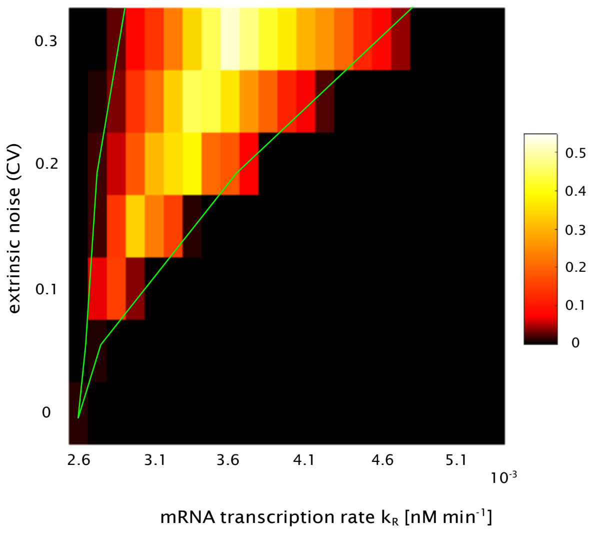

FIG. S5: Bimodality amplitude phase diagram. Phase diagram of the bimodality amplitude of the mRNA distribution as a function of the mRNA transcription rate k_R and of the extrinsic noise level.

The parameters here used are the following: $k_P = 6.0$ min^{-1}, $g_S = 1.2 \times 10^{-2}$ min^{-1}, $g_P = 1.2 \times 10^{-2}$ min^{-1}, $g_R = 2.4 \times 10^{-2}$ min^{-1}, $g = 3.0 \times 10^{-2}$ nM^{-1} min^{-1}, $\alpha = 0.5$. Target mRNA transcription rate is one of the control parameters and ranges from $k_R = 2.6 \times 10^{-3}$ nM min^{-1} to $k_R = 5.1 \times 10^{-3}$ nM min^{-1}. Extrinsic noise is tuned by varying the standard deviation of the distribution with mean $\overline{k_S} = 1.2 \times 10^{-3}$ nM min^{-1} from which miRNA transcription rates are picked. The standard deviation ranges from $\sigma = 0$ nM min^{-1} (no extrinsic noise) to $\sigma = 3.6 \times 10^{-4}$ nM min^{-1}. This phase diagram was obtained by interpolating the single distributions obtained from numerical simulations as described in Sec. V. The green line represents the separation between bimodal and unimodal regions as shown in the Main Text.
