# Supplementary material for: On the role of extrinsic noise in microRNA-mediated bimodal gene expression
Source: PLoS Comput Biol. 2018 Apr 17;14(4):e1006063. doi: 10.1371/journal.pcbi.1006063 (PMC5922620; doi:10.1371/journal.pcbi.1006063)
Supplement: S6 Fig — (A) Example of two average mRNA profiles, for a regulated (orange) and an unregulated (blue) mRNA. (B) Same profile as panel A but with the curves shifted upwards by an arbitrary offset of 10 mRNA molecules. (C) Fold repression (ratio of blue to orange curve from panel A) without the offset. (D) Fold repression (ratio of blue to orange curve from panel B) with the offset. (E) Comparison of the plots of fold repression with offset (from panel D) and without offset (from panel C). The parameters here used are the following: gS = 1.2 × 10−2 min−1, gR = 2.4 × 10−2 min−1, g = 3.0 × 101 nM−1 min−1, kS = 7.1 × 10^−4^ nM min−1, α = 0.5. kR ranges from 0 nM min−1 to 4.8 × 10^−3^ nM min−1. (PDF) [file pcbi.1006063.s007.pdf]

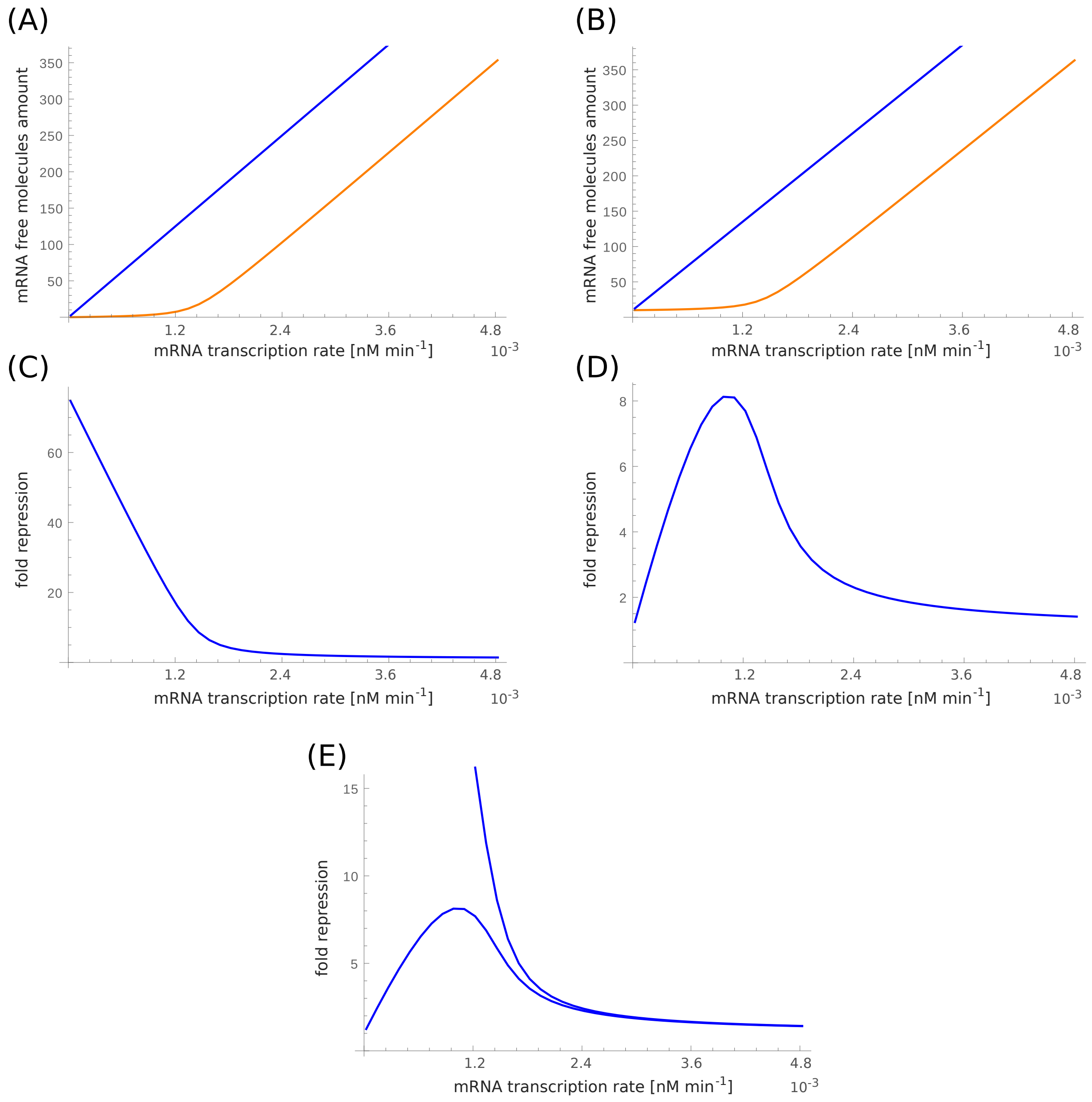

FIG. S6: The role of the offset. (A) Example of two average mRNA profiles, for a regulated (orange) and an unregulated (blue) mRNA. (B) Same profile as panel A but with the curves shifted upwards by an arbitrary offset of 10 mRNA molecules. (C) Fold repression (ratio of blue to orange curve from panel A) without the offset. (D) Fold repression (ratio of blue to orange curve from panel B) with the offset. (E) Comparison of the plots of fold repression with offset (from panel D) and without offset (from panel C). The parameters here used are the following: $g_S = 1.2 \times 10^{-2}$ min^{-1}, $g_R = 2.4 \times 10^{-2}$ min^{-1}, $g = 3.0 \times 10^{1}$ nM^{-1} min^{-1}, $k_S = 7.1 \times 10^{-4}$ nM min^{-1}, $\alpha = 0.5$. k_R ranges from 0 nM min^{-1} to 4.8×10^{-3} nM min^{-1}.
